# Real‐World Dual Antiplatelet Therapy Use Exceeds Randomized Trials Boundaries With Possible Safety Issues in Patients With Large Artery Atherosclerosis—Insights From the READAPT Study

**DOI:** 10.1111/ene.70163

**Published:** 2025-04-23

**Authors:** Eleonora De Matteis, Federico De Santis, Matteo Foschi, Michele Romoli, Tiziana Tassinari, Valentina Saia, Silvia Cenciarelli, Chiara Bedetti, Chiara Padiglioni, Bruno Censori, Valentina Puglisi, Luisa Vinciguerra, Maria Guarino, Valentina Barone, Marialuisa Zedde, Ilaria Grisendi, Ilaria Maestrini, Maria Rosaria Bagnato, Marco Petruzzellis, Domenico Maria Mezzapesa, Pietro Di Viesti, Vincenzo Inchingolo, Manuel Cappellari, Mara Zenorini, Paolo Candelaresi, Vincenzo Andreone, Giuseppe Rinaldi, Alessandra Bavaro, Anna Cavallini, Stefan Moraru, Maria Grazia Piscaglia, Valeria Terruso, Marina Mannino, Alessandro Pezzini, Giovanni Frisullo, Francesco Muscia, Maurizio Paciaroni, Maria Giulia Mosconi, Andrea Zini, Ruggiero Leone, Carmela Palmieri, Letizia Maria Cupini, Michela Marcon, Rossana Tassi, Enzo Sanzaro, Cristina Paci, Giovanna Viticchi, Daniele Orsucci, Anne Falcou, Susanna Diamanti, Roberto Tarletti, Patrizia Nencini, Eugenia Rota, Federica Nicoletta Sepe, Delfina Ferrandi, Luigi Caputi, Gino Volpi, Salvatore La Spada, Mario Beccia, Claudia Rinaldi, Vincenzo Mastrangelo, Francesco Di Blasio, Paolo Invernizzi, Giuseppe Pelliccioni, Maria Vittoria De Angelis, Laura Bonanni, Giampietro Ruzza, Emanuele Alessandro Caggia, Monia Russo, Agnese Tonon, Maria Cristina Acciarri, Sabrina Anticoli, Cinzia Roberti, Giovanni Manobianca, Gaspare Scaglione, Francesca Pistoia, Alberto Fortini, Antonella De Boni, Alessandra Sanna, Alberto Chiti, Leonardo Barbarini, Marcella Caggiula, Maela Masato, Massimo Del Sette, Francesco Passarelli, Maria Roberta Bongioanni, Danilo Toni, Stefano Ricci, Simona Sacco, Raffaele Ornello, the READAPT Study Group, Maurizio Acampa, Maria Cristina Acciarri, Paola Ajdinaj, Sergio Altomare, Chiara Alessi, Vincenzo Andreone, Stefania Martina Angelocola, Sabrina Anticoli, Federica Assenza, Maria Rosaria Bagnato, Leonardo Barbarini, Chiara Bassi, Valentina Barone, Maraia Cristina Baruffi, Alessandra Bavaro, Mario Beccia, Chiara Bedetti, Simone Bellavia, Simone Beretta, Giorgia Bernabè, Leonardo Biscetti, Novella Bonaffini, Laura Bonanni, Maria Roberta Bongioanni, Gian Luca Bruzzone, Martina Caccamo, Marcella Caggiula, Valentina Cameriere, Paolo Candelaresi, Alessandro Canessa, Luigi Caputi, Nicoletta Giuseppa Caracciolo, Patrizio Cardinali, Anna Cavallini, Emanuele Alessandro Caggia, Roberto Cappellani, Manuel Cappellari, Silvia Cenciarelli, Bruno Censori, Alberto Chiti, Letizia Maria Cupini, Maria Vittoria De Angelis, Antonella De Boni, Chiara De Fino, Cristina De Luca, Antonio De Mase, Eleonora De Matteis, Manuela De Michele, Federico De Santis, Massimo Del Sette, Francesco Di Blasio, Ivo Giuseppe De Franco, Anna Di Giovanni, Filomena Di Lisi, Pietro Di Viesti, Susanna Diamanti, Marina Diomedi, Anna Falcou, Claudia Faini, Ciro Alberto Fasolino, Delfina Ferrandi, Carlo Ferrarese, Thomas Fleetwood, Alberto Fortini, Matteo Foschi, Giovanni Matteo Fratta, Giovanni Frisullo, Antonio Genovese, Serena Gallo Cassarino, Debora Galotto, Maurizio Giorelli, Alessia Giossi, Ilaria Grisendi, Maria Guarino, Vincenzo Inchingolo, Paolo Invernizzi, Salvatore La Spada, Ruggiero Leone, Federica Letteri, Enrico Maria Lotti, Ilaria Maestrini, Marina Mannino, Giovanni Manobianca, Michela Marcon, Maela Masato, Vincenzo Mastrangelo, Chiara Menichetti, Elisabetta Menegazzo, Domenico Maria Mezzapesa, Daniela Monaco, Stefan Moraru, Maria Giulia Mosconi, Francesco Muscia, Federica Naldi, Serena Nannucci, Patrizia Nencini, Raffaele Ornello, Daniele Orsucci, Cristina Paci, Maurizio Paciaroni, Chiara Padiglioni, Carmela Palmieri, Silvia Paolucci, Giulio Papiri, Rosario Pascarella, Francesco Passarelli, Giuseppe Pelliccioni, Francesco Perini, Marco Petruzzellis, Francesca Pistoia, Eleonora Potente, Emanuele Puca, Valentina Puglisi, Pietro Querzani, Stefano Ricci, Maria Chiara Ricciardi, Claudia Rinaldi, Giuseppe Rinaldi, Annalisa Rizzo, Cinzia Roberti, Michele Romoli, Eugenia Rota, Monia Russo, Giampietro Ruzza, Elisa Sacchini, Simona Sacco, Valentina Saia, Alessandra Sanna, Enzo Sanzaro, Davide Sassos, Gaspare Scaglione, Irene Scala, Alessandro Pezzini, Eleonora Sgarlata, Emanuele Spina, Roberto Tarletti, Rossana Tassi, Tiziana Tassinari, Valeria Terruso, Pierluigi Tocco, Danilo Toni, Agnese Tonon, Laura Tudisco, Martina Valente, Maria Grazia Vittorini, Gloria Valcamonica, Luisa Vinciguerra, Marco Vista, Giovanna Viticchi, Gino Volpi, Marialuisa Zedde, Mara Zenorini, Andrea Zini, Cecilia Zivelonghi, Antonio Zito

**Affiliations:** ^1^ Department of Brain Sciences Imperial College London London UK; ^2^ Department of Biotechnological and Applied Clinical Sciences University of L'Aquila L'Aquila Italy; ^3^ Department of Neuroscience Maurizio Bufalini Hospital, AUSL Romagna Cesena Italy; ^4^ Department of Neurology Santa Corona Hospital Pietra Ligure Italy; ^5^ Department of Neurology Città di Castello Hospital Città di Castello Italy; ^6^ Department of Neurology ASST Crema Hospital Cremona Italy; ^7^ IRCCS Istituto Delle Scienze Neurologiche di Bologna Bologna Italy; ^8^ Neurology Unit, Stroke Unit Azienda Unità Sanitaria Locale‐IRCCS di Reggio Emilia Reggio Emilia Italy; ^9^ Stroke Center Tor Vergata University Hospital Rome Italy; ^10^ Stroke Unit, Department of Neurology “F. Puca” AOU Consorziale Policlinico Bari Italy; ^11^ Department of Neurology Fondazione Casa Sollievo Della Sofferenza San Giovanni Rotondo Italy; ^12^ Department of Neuroscience Azienda Ospedaliera Universitaria Integrata Verona Verona Italy; ^13^ Stroke Unit, Department of Neurology AORN Antonio Cardarelli Naples Italy; ^14^ Department of Neurology Di Venere Hospital Bari Italy; ^15^ UO Neurologia D'urgenza e Stroke Unit IRCCS Mondino Foundation Pavia Italy; ^16^ Department of Neuroscience S. Maria Delle Croci Hospital, AUSL Romagna Ravenna Italy; ^17^ Department of Neurology AOOR Villa Sofia‐Cervello Palermo Italy; ^18^ Department of Medicine and Surgery University of Parma Parma Italy; ^19^ Stroke Care Program, Department of Emergency Parma University Hospital Parma Italy; ^20^ Neuroscienze, Organi di Senso e Torace Fondazione Policlinico Universitario Agostino Gemelli Rome Italy; ^21^ Department of Neurology ASST‐Ovest Milanese Legnano Italy; ^22^ Department of Neurosciences and Rehabilitation University of Ferrara Ferrara Italy; ^23^ Stroke Unit, Department of Internal and Cardiovascular Medicine University Hospital Santa Maria Della Misericordia Perugia Italy; ^24^ Department of Neurology and Stroke Center, Maggiore Hospital IRCCS Istituto Delle Scienze Neurologiche di Bologna Bologna Italy; ^25^ Department of Neurology and Stroke Unit “M. R. Dimiccoli” General Hospital Barletta Italy; ^26^ Stroke Unit, Department of Neurology E. Agnelli Hospital Pinerolo Italy; ^27^ Stroke Unit, Department of Neurology S. Eugenio Hospital Rome Italy; ^28^ Department of Neurology Cazzavillan Hospital Arzignano Vicenza Italy; ^29^ Urgency and Emergency Department Azienda Ospedaliera Universitaria Senese Siena Italy; ^30^ Department of Neurology Umberto I Hospital Siracusa Italy; ^31^ UOC Neurologia Ospedale “Madonna del Soccorso” San Benedetto del Tronto Italy; ^32^ Experimental and Clinical Medicine Department Marche Polytechnic University Ancona Italy; ^33^ Unit of Neurology‐San Luca Hospital Lucca and Castelnuovo Garfagnana Italy; ^34^ Stroke Unit, Emergency Department Policlinico Umberto I Hospital Rome Italy; ^35^ Department of Neurology Fondazione IRCCS San Gerardo Dei Tintori Monza Italy; ^36^ Stroke Unit, SCDU Neurologia Azienda Ospedaliero‐Universitaria “Maggiore Della Carità” Novara Italy; ^37^ Stroke Unit Careggi University Hospital Florence Italy; ^38^ Department of Neurology San Giacomo Hospital Novi Ligure Italy; ^39^ Stroke Unit, Department of Neurology SS. Biagio e Arrigo Hospital Alessandria Italy; ^40^ Department of Cardiocerebrovascular Diseases Neurology‐Stroke Unit‐ASST Ospedale Maggiore di Crema Crema Italy; ^41^ Department of Neurology San Jacopo Hospital Pistoia Italy; ^42^ Department of Neurology Antonio Perrino Hospital Brindisi Italy; ^43^ Department of Neurology Sant'andrea Hospital Rome Italy; ^44^ Neurology Unit “Infermi” Hospital, AUSL Romagna Rimini Italy; ^45^ Stroke Unit “S.Spirito” Hospital Pescara Italy; ^46^ Departiment of Neurology Istituto Ospedaliero Fondazione Poliambulanza Brescia Italy; ^47^ Department of Neurology INRCA Ancona Italy; ^48^ Stroke Unit, Department of Neurology SS Annunziata Hospital Chieti Italy; ^49^ Dipartimento di Medicina e Scienze Dell'Invecchiamento, Università G. D'Annunzio di Chieti‐Pescara E Clinica Neurologica e Stroke Unit Ospedale Clinicizzato SS. Annunziata di Chieti Chieti Italy; ^50^ Department of Neurology Civil Hospital Cittadella Italy; ^51^ Neurology Unit, Cardio‐Neuro‐Vascular Department Giovanni Paolo II Hospital Ragusa Italy; ^52^ Department of Neurology St Misericordia Hospital Rovigo Italy; ^53^ Department of Neurology Ospedale Civile SS. Giovanni e Paolo Venice Italy; ^54^ Department of Neurology A. Murri Fermo Hospital Fermo Italy; ^55^ Stroke Unit Azienda Ospedaliera San Camillo Rome Italy; ^56^ Department of Neurology San Filippo Neri Hospital Rome Italy; ^57^ Department of Neurology General Regional Hospital “F. Miulli” Acquaviva Delle Fonti Italy; ^58^ Internal Medicine San Giovanni di Dio Hospital Florence Italy; ^59^ Department of Neuroscience San Bortolo Hospital Vicenza Italy; ^60^ Stroke Unit AOU Sassari Sassari Italy; ^61^ Unit of Neurology Apuane Hospital Massa Italy; ^62^ Department of Neurology Vito Fazi Hospital Lecce Italy; ^63^ Department of Neurology Mirano Hospital Mirano Italy; ^64^ Department of Neuroscience IRCCS Ospedale Policlinico San Martino Genoa Italy; ^65^ Department of Neurology Fatebenefratelli Hospital Rome Italy; ^66^ Department of Neurology SS Annunziata Hospital Savigliano Italy; ^67^ Department of Human Neurosciences University of Rome La Sapienza Rome Italy; ^68^ Coordinatore Comitato Scientifico ISA‐AII Italy

**Keywords:** dual antiplatelet therapy, large artery atherosclerosis, minor ischemic stroke, real world, TIA

## Abstract

**Background and Aim:**

According to randomized controlled trials (RCTs), dual antiplatelet therapy (DAPT) is more effective for secondary prevention of ischemic events attributable to large artery atherosclerosis (LAA) than other mechanisms. We investigated whether real‐world application may impact DAPT effectiveness and safety in the REAl‐life study on short‐term Dual Antiplatelet treatment in Patients with ischemic stroke or Transient ischemic attack (READAPT, NCT05476081).

**Methods:**

READAPT was an observational multicenter study including patients with minor ischemic stroke or TIA treated with short‐term DAPT. At 90 days, we assessed primary effectiveness (ischemic recurrence, severe bleeding, or vascular death) and safety (severe to moderate bleeding) outcomes. We explored associations between LAA and outcomes using Cox regression. Within patients with and without LAA, outcomes were compared between subgroups based on age, NIHSS score (for ischemic stroke patients), ABCD^2^ score (for TIA patients), presence and number of MRI acute lesions, and DAPT regimen characteristics.

**Results:**

Among 1920 analyzed patients (of 2278 enrolled), 452 had LAA. Unlike RCTs, 21.2% of patients with LAA had NIHSS > 5, and 48.2% received DAPT > 30 days. Patients with LAA had higher bleeding rates (3.5% vs. 2.1%, *p* = 0.004), primarily hemorrhagic infarctions and moderate bleeding, than those without LAA. However, primary effectiveness outcomes were similar (4.9% vs. 3.5%, *p* = 0.201) between the groups. In patients with LAA, prolonged DAPT (> 21 days), multiple MRI lesions, age ≥ 65, and loading doses increased bleeding risk.

**Conclusions:**

The real‐world DAPT use in patients with LAA exceeds RCTs boundaries with possible drawbacks on treatment safety.

## 1 Introduction

Large‐artery atherosclerosis (LAA), whether extracranial or intracranial, accounts for up to 15% of cerebrovascular ischemic events [1, 2]. Over recent decades, the risk of ischemic recurrences following an LAA‐related event has significantly decreased due to advances in secondary prevention strategies [3]. However, the short‐term risk of ischemic recurrences remains higher after an LAA event than from other causes [3]. The 90‐day risk is estimated to be around 13% after a minor stroke or high‐risk TIA in the presence of ≥ 50% extracranial carotid stenosis [4] or symptomatic intracranial stenosis [5]. Randomized controlled trials (RCTs) have demonstrated the superiority of dual antiplatelet therapy (DAPT) compared with single antiplatelet therapy (SAPT) after LAA minor strokes or high‐risk TIAs [6, 7]. This benefit is evident even when DAPT is started within 72 h from symptom onset [8].

Overall, RCTs support the use of DAPT for secondary prevention of LAA cerebrovascular ischemic event [6, 7, 8, 9], but they exhibited heterogeneity and some limitations. While DAPT has been associated with a reduced risk of ischemic recurrences compared to SAPT in patients with LAA, subgroup analyses from CHANCE and THALES failed to demonstrate a significant interaction between DAPT and LAA [5, 7]. Additionally, treatment benefits/risks compared with other causes remain unclear. THALES and ATAMIS subgroup analyses suggested greater DAPT benefit in patients with atherosclerotic events [6, 7]. However, in THALES, the definition of ipsilateral atherosclerosis differs from that used in the other RCTs, as do the definitions of minor stroke and high‐risk TIA [6, 7]. Lastly, real‐world use of DAPT extends beyond RCT settings [10], with potential benefits observed in patients outside RCT boundaries for treatment regimens and index event characteristics [11].

We hypothesized that, in the real world, physicians may prescribe DAPT to patients not strictly meeting RCTs definitions for LAA and non‐LAA minor ischemic stroke or high‐risk TIA. The characteristics of patients and their index event, as well as deviations from RCTs DAPT regimens, could influence treatment effectiveness and safety. To evaluate these hypotheses, we conducted this subgroup analysis of the REAl‐life study on short‐term Dual Antiplatelet treatment in Patients with ischemic stroke or Transient ischemic attack (NCT05476081).

## 2 Methods

The READAPT (NCT05476081) methods have been summarized elsewhere [10, 11]. Briefly, this was an observational prospective multicenter real‐world study endorsed by the Italian Stroke Association (ISA‐AII). The study was conducted according to the Strengthening the Reporting of Observational Studies in Epidemiology (STROBE) guidelines [12] and was approved with the number 03/2021 by The Internal review Board of the University of L'Aquila—the coordinating center—in February 2021. We included patients treated with DAPT at one of the 64 participating Italian centers (Table S8) between February 2021 and February 2023. Patients were included shortly after the index event (i.e., baseline) and had a 90‐day follow‐up, which concluded with a face‐to‐face or remotely end of study visit.

### 2.1 Study Inclusion and Exclusion Criteria

We included inpatients or outpatients with non‐cardioembolic minor ischemic stroke or high‐risk TIA according to the World Health Organization (WHO) time‐based definition, older than 18 years, and receiving short‐term DAPT. All patients or their proxies had to sign an informed consent to join the study. We excluded patients treated with DAPT because of endovascular stenting procedures and those who were already enrolled in interventional RCTs on stroke prevention. As elsewhere mentioned [10, 11], no NIHSS and ABCD^2^ threshold were established but we encouraged physicians to adhere to national and international guidelines [13, 14, 15, 16].

For this analysis, we divided the cohort into patients with LAA and those without (non‐LAA). The cause of the index event was adjudicated by local physicians and centrally reviewed. We recommend physicians to strictly adhere to the Trial of Org 10,172 in Acute Stroke Treatment (TOAST) definition of LAA events—either significant (> 50%) stenosis or occlusion of a major brain artery or branch cortical artery, presumably due to atherosclerosis [17]. However, due to the observational design of the study, few events were classified as LAA when the ipsilateral atherosclerotic plaque caused less than 50% stenosis but was unstable, and no other plausible causes were identified. Intracranial CT or MR angiography (CTA or MRA) was mandatory for the diagnosis of intracranial atherosclerosis, whereas various examination methods, including ultrasound, CTA, or MRA, were permitted for assessing extracranial stenosis.

### 2.2 Study Procedures and Data Collection

Similarly to minor ischemic stroke and high‐risk TIA definitions, we did not provide physicians strict DAPT regimen indications. Indeed, they chose DAPT dosage, duration, and the type of antiplatelet prescribed at the end of the DAPT period case by case according to the best clinical practice and guidelines [13, 14, 15, 16].

Anonymized data were collected on an electronic database—created with Research Electronic Data Capture (REDCap) software [18, 19]—that was hosted at University of L'Aquila. The study staff regularly checked the integrity of data collection and completeness of follow‐up as previously reported [10, 11].

### 2.3 Outcomes

The primary effectiveness outcome was a composite of new stroke events (ischemic or hemorrhagic) or death due to vascular causes at 90 days. The primary safety outcome was a moderate‐to‐severe bleeding at 90 days. Secondary outcomes were ischemic stroke (i.e., ischemic stroke or TIA), intracerebral hemorrhage (ICH), subarachnoid hemorrhage, other intracranial hemorrhage (i.e., subdural hematoma, epidural hematoma, or other), symptomatic and asymptomatic hemorrhagic infarction defined according to the Heidelberg Bleeding Classification [20], myocardial infarction, vascular death, non‐vascular death, any hospitalization, disability measured by the modified Rankin Scale (mRS), cause of DAPT discontinuation (i.e., adverse events, lack of compliance, other), severe bleeding, moderate bleeding, and mild bleeding—defined according to the Global Utilization of Streptokinase and Tissue Plasminogen Activator for Occluded Coronary Arteries (GUSTO) trial [21]. Secondary composite outcomes were any bleeding (i.e., any of severe, moderate, or mild bleeding) and any death (i.e., any of death due to vascular or non‐vascular causes).

We reported the outcomes in patients with LAA and with non‐LAA. Within these two main groups, we reported the rates of primary effectiveness outcome and any bleeding in pre‐specified subgroups based on demographics and disease characteristics that could potentially impact treatment effectiveness and safety. These subgroups included age > or ≤ 65 years, body mass index (BMI) < or ≥ 30, ischemic strokes with NIHSS score ≤ 3 or > 3, < or ≥ 2 MRI acute lesions when an MRI scan was performed, TIA with ABCD^2^ score ≤ or > 4, prior antiplatelet therapy, revascularization procedure, DAPT loading dose, time to DAPT start ≤ or > 24 h, DAPT duration ≤ or > 21 days, early DAPT discontinuation. Lastly, within patients with LAA, we reported the outcomes in those with ipsilateral extracranial carotid stenosis < 50% and ≥ 70%.

### 2.4 Statistical Analysis

We performed intention‐to‐treat analyses in patients who completed the follow‐up or had an outcome event within 90 days. We excluded patients who had a follow‐up visit prior to 80 days, and those who underwent stenting procedures or switched to anticoagulation therapy during the follow‐up. If multiple outcome events occurred, only the first was retained.

We reported descriptive statistics about demographics, characteristics of the index event, and outcome events. Categorical data were reported as number and percentage. Continuous data had non‐normal distribution at the Kolmogorov–Smirnov test and were reported as median and interquartile range (IQR). Categorical and continuous data were compared across patients' groups through the chi‐squared and Mann–Whitney *U* tests, respectively. Kaplan–Meier curves and log‐rank test were used to represent and compare the cumulative risk of primary effectiveness and any bleeding in patients with LAA and non‐LAA. Univariate and multivariate Cox regressions were used to identify factors associated with the primary effectiveness and any bleeding in the entire study cohort. Only covariates with *p* < 0.1 were included in the multivariate model. Statistics and graphs were performed through R version 4.4.2. and GraphPad version 10.

## 3 Results

**FIGURE 1:**
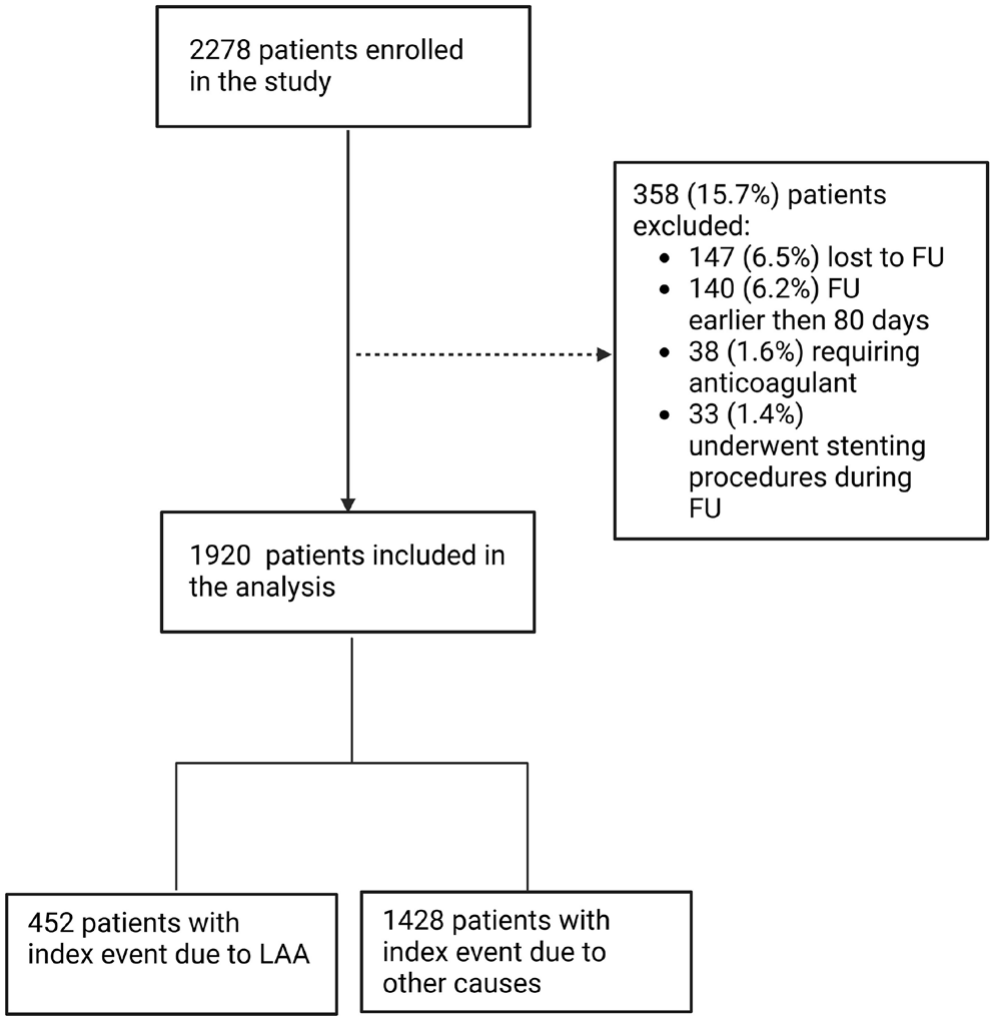
Consolidated standards of reporting trials diagram of enrolment in the study. LAA, large‐artery atherothrombosis; FU, follow‐up.

Of the 2278 patients originally enrolled between February 2021 and February 2023, 1920 (84.3%) had follow‐up data and were included in this subgroup analysis (Figure 1). According to the cause of the index event, 452 (23.5%) patients had an event attributable to LAA.

### 3.1 Baseline Characteristics

#### 3.1.1 Demographics and Comorbidities

Compared with patients receiving DAPT after a non‐LAA index event, patients with LAA were older (median age 73, IQR 65–79 vs. 72, IQR 61–79 *p* = 0.011) and more frequently male (318, 70.2% vs. 917, 63.8%, *p* = 0.011). Patients with LAA also had more frequent vascular comorbidities such as hypertension (375, 83.0% vs. 1152, 78.5%, *p* = 0.039), diabetes mellitus (140, 31.0% vs. 381 26.0%, *p* = 0.036), dyslipidemia (292, 64.6% vs. 868 59.1%, *p* = 0.037), hypertriglyceridemia (114, 25.2% vs. 284 19. 3%, *p* = 0.007), and peripheral chronic obliterative arteriopathy (39, 8.6% vs. 71, 4.8%, *p* = 0.002) compared with those with non‐LAA. Treatment with antiplatelet agents before the index event was similar between the groups (187, 41.4% vs. 596, 40.6%, *p* = 0.770; Table 1).

**TABLE 1:** Demographics and characteristics of the index event among patients with large‐artery atherosclerosis and those with non‐large‐artery atherosclerosis.

Characteristics	LAA^a^ (*N* = 452)	Non‐LAA^a^ (*N* = 1468)	*p*
Age, years, median (IQR)	73 (65–79)	72 (61–79)	**0.011**
Female gender, *N* (%)	134 (29.6)	551 (36.2)	**0.011**
Caucasian, *N* (%)	444 (98.2)	1433 (97.6)	0.619
BMI, median (IQR)	26 (24–28)	26 (23–28)	0.066
Current smoker, *N* (%)	131 (29.0)	361 (24.6)	0.837
Hypertension, *N* (%)	375 (83.0)	1152 (78.5)	**0.039**
Diabetes mellitus, *N* (%)	140 (31.0)	381 (26.0)	**0.036**
Dyslipidemia, *N* (%)	292 (64.6)	868 (59.1)	**0.037**
Hypertriglyceridemia	114 (25.2)	284 (19.3)	**0.007**
Previous ischemic event (TIA or ischemic stroke), *N* (%)	103 (22.8)	263 (17.9)	0.508
Previous intracerebral hemorrhage, *N* (%)	3 (0.6)	12 (0.8)	0.745
Myocardial infarction, *N* (%)	51 (11.5)	131 (8.9)	0.102
Angina, *N* (%)	20 (4.4)	42 (2.9)	0.1
Congestive heart failure, *N* (%)	16 (3.5)	42 (2.9)	0.461
Peripheral chronic obliterative arteriopathy, *N* (%)	39 (8.6)	71 (4.8)	**0.002**
Use of antiplatelet prior to the index event, *N* (%)	187 (41.4)	596 (40.6)	0.77
Symptom duration, *N* (%)			0.125
> 24 h	329 (72.8)	1013 (69.0)	
< 24 h	123 (27.2)	455 (31.0)	
Lesions at neuroimaging, *N* (%)			**0.002**
Yes	334 (73.9)	968 (65.9)	
No	118 (26.1)	500 (34.1)	
ABCD^2^ score in patients with qualifying TIA, median (IQR)	5 (4–6)	4 (4–5)	0.179
ABCD^2^ < 4, *N* (%)	26 (21.1)	124 (22.7)	0.908
ABCD^2^ < 6 and no LAA, *N* (%)	102 (82.9)	371 (68.0)	0.133
NIHSS score in patients with qualifying ischemic stroke, median (IQR) and [range]	3 (2–5)	3 (2–4)	**0.001**
NIHSS > 3, *N* (%)	150 (45.6)	325 (32.0)	**0.019**
NIHSS > 5, *N* (%)	70 (21.2)	89 (8.7)	**0.001**
mRS baseline, median (IQR)	0 (0–0)	0 (0–0)	0.111
Time to DAPT start, *N* (%)			0.096
< 12 h	146 (32.3)	496 (33.8)	
12–24 h	135 (29.9)	484 (33.0)	
25–48 h	92 (20.4)	298 (20.3)	
> 48 h	79 (17.5)	190 (12.9)	
Type of DAPT, *N* (%)			0.199
Aspirin/Clopidogrel	372 (99.2)	1540 (99.8)	
Aspirin/Ticagrelor	3 (0.8)	5 (0.2)	
Loading dose, *N* (%)	244 (54.0)	677 (46.1)	0.97
Aspirin	152 (33.6)	400 (27.2)	**0.009**
Clopidogrel	150 (33.2)	553 (37.7)	0.084
Ticagrelor	3 (0.7)	2 (0.1)	0.465
Revascularization procedures, *N* (%)	98 (19.7)	238 (16.2)	0.085
DAPT duration, median (IQR)	30 (21–90)	21 (21–30)	**0.001**
DAPT duration < 21 days, *N* (%)	37 (8.2)	87 (5.9)	**0.001**
DAPT duration 21–30 days, *N* (%)	197 (43.6)	1059 (72.4)	
DAPT duration 30–90 days, *N* (%)	218 (48.2)	322 (21.9)	
DAPT discontinuation before expected completion, *N* (%)			0.178
Adverse events	11 (2.4)	11 (0.7)	
Lack of compliance	3 (0.6)	12 (0.8)	
Other	21 (4.6)	30 (2.0)	

*Note:* Statistical significance was defined as a *p*‐value < 0.05 and marked in bold. Abbreviations: BMI, body mass index; DAPT, dual antiplatelet therapy; h, hours; IQR, interquartile range; LAA, large‐artery atherosclerosis; LD, loading dose; mRS, modified Rankin scale; *N*, number; NIHSS, National Institutes of Health Stroke Scale; TIA, transient ischemic attack.

^a^ All patients were evaluable for the analysis.

#### 3.1.2 Vascular Characteristics and Incidence of Carotid Stenosis in Patients With LAA

**FIGURE 2:**
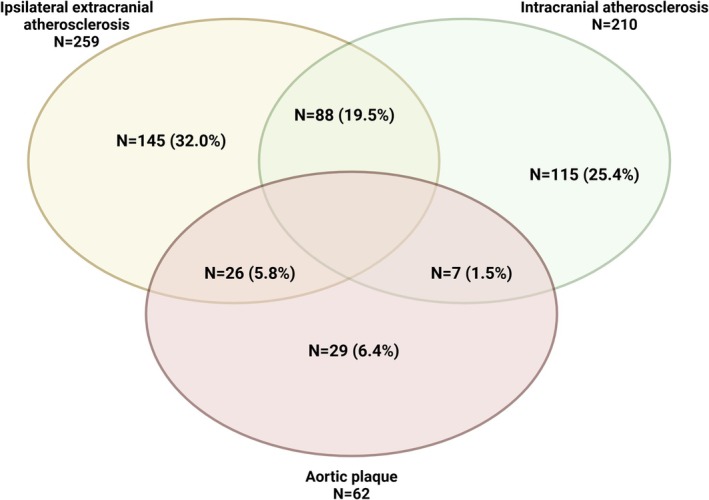
Presence of ipsilateral extracranial stenosis, symptomatic intracranial stenosis, and/or aortic plaque in patients with an index event attributable to large‐artery atherosclerosis. *N*, number.

Among patients with LAA, 259 (57.3%) had ipsilateral extracranial atherosclerosis, which in 183 (70.6%) of the cases affected the carotid district; 210 (46.4%) patients had intracranial stenosis, and 62 (13.7%) had an aortic plaque (Figure 2). Notably, 88 (19.5%) patients had both extra‐ and intracranial carotid stenosis ipsilateral to symptom location. Additionally, 36 (23.2%) underwent urgent carotid endarterectomy after the index event.

**FIGURE 3:**
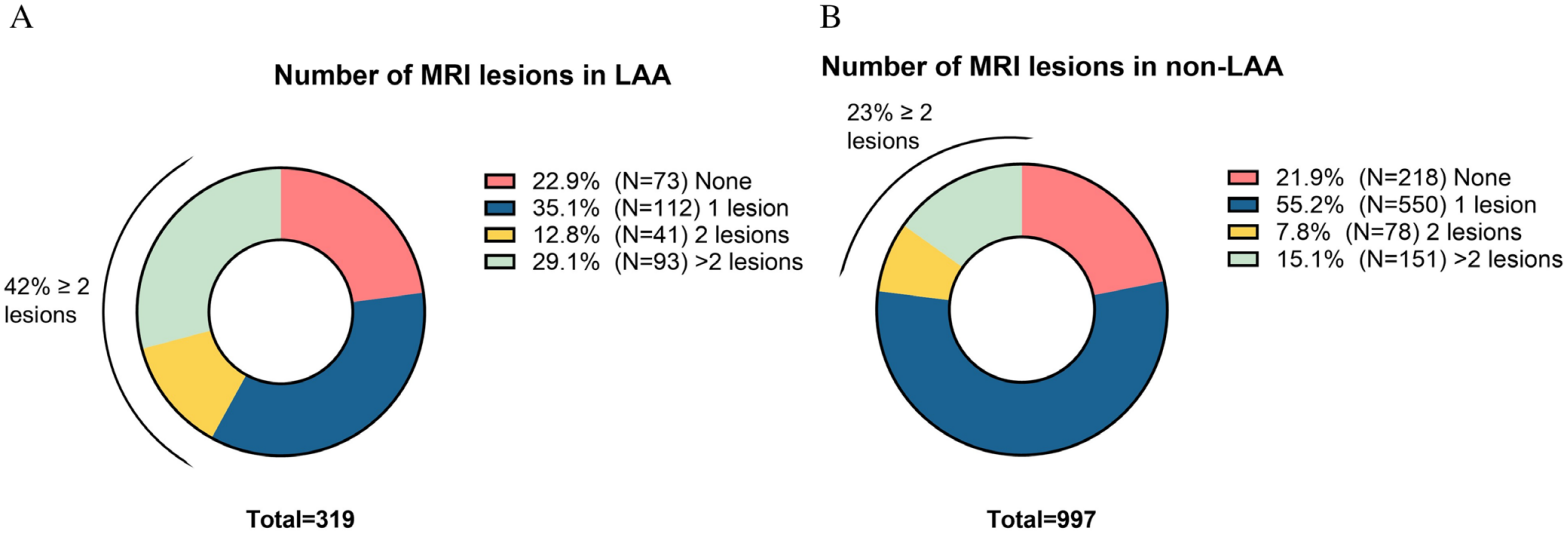
Number of MRI lesions in patients with large‐artery atherosclerosis (A) and with non‐large‐artery atherosclerosis (B). LAA, large‐artery atherosclerosis, *N*, number.

#### 3.1.3 Characteristics of the Index Event and DAPT Regimen

Similarly to non‐LAA counterpart, most patients with LAA received DAPT after an ischemic stroke (329, 72.8% vs. 1013, 69%, *p* = 0.125). In patients with LAA, the severity of the ischemic stroke was significantly higher: 150 (45.6%) patients with LAA presented with an NIHSS > 3 and 118 (26.1%) with an NIHSS > 5 compared with 325 (32.0%, *p* = 0.019) and 89 (8.7%, *p* = 0.001) patients with non‐LAA events, respectively. Additionally, median DAPT duration was significantly longer in patients with LAA (30 days, IQR 21–90) than in those with non‐LAA (21 days, IQR 21–30, *p* ≤ 0.001) with 48.2% patients continuing the therapy after 30 days.

Considering neuroimaging characteristics, patients with LAA more frequently had an acute lesion (334, 73.9% vs. 968, 65.9% *p* = 0.002) at either CT or MRI scan performed in the first days after the index event. Among the 1316 patients who underwent an MRI scan (319 LAA and 997 non‐LAA), significantly more patients with LAA had ≥ 2 acute MRI lesions compared with patients with non‐LAA (134, 42.0% vs. 193, 23.0%; *p* < 0.001) (Figure 3). In patients with LAA, multiple lesions were present in 128 (95.5%) of cases in the same vascular territory. Conversely, patients with and without LAA had similar rates of neuroimaging findings suggestive of cerebral small vessel disease (cSVD) (Table S1). Specifically, the rates of cortical superficial siderosis were comparable between the groups (5 patients, 1.1%, with LAA and 12, 1.2%, with non‐LAA, *p* = 0.616), as were the rates of microbleeds (12 patients, 2.7%, with LAA and 58, 5.8%, with non‐LAA, *p* = 0.154). The two groups also exhibited similar Fazekas scores for deep white matter (median of 2, IQR 1–3, for both groups, *p* = 0.822) and periventricular white matter (median of 2, IQR: 1–2 for both groups, *p* = 0.501).

### 3.2 Outcomes

#### 3.2.1 DAPT Effectiveness and Safety in Patients With LAA and Non‐LAA

**FIGURE 4:**
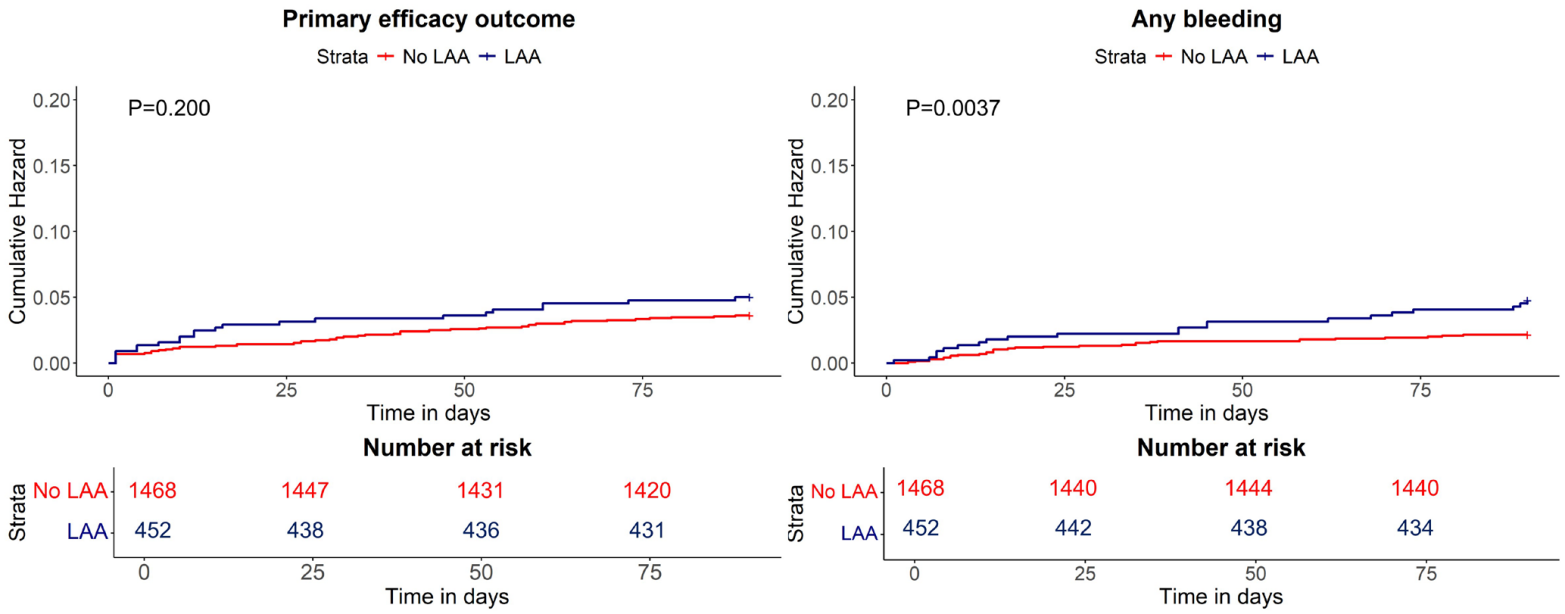
Primary effectiveness outcome and any bleeding in patients with large‐artery atherosclerosis and with non‐large‐artery atherosclerosis. LAA, large‐artery atherosclerosis.

DAPT effectiveness was similar between patients with and without LAA. Out of the 74 primary effectiveness outcomes, 22 (4.9%) occurred in patients with LAA and 52 (3.5%) in those with non‐LAA (*p* = 0.201) (Table 2). Accordingly, the survival analyses showed a similar cumulative risk of primary effectiveness outcome over the 90‐day follow‐up (*p* = 0.200) between the two groups (Figure 4). Ischemic recurrences accounted for most of the primary effectiveness outcomes in both groups (19, 4.2% in LAA vs. 45, 3.1% in non‐LAA, *p* = 0.239); one vascular death (0.2%) occurred in patients with LAA and 2 (0.1%) in those with non‐LAA (*p* = 0.351) (Table 2). The sole factor associated with a lower risk of primary effectiveness outcome was the female gender (HR 0.56, 95% CI 0.32–0.96, *p* = 0.03) (Table S2).

**TABLE 2:** Outcomes in the two subgroups: Patients with large‐artery atherosclerosis and those with non‐large‐artery atherosclerosis.

Outcome	LAA (*N* = 452)	Non‐LAA (*N* = 1428)	*p*
Primary effectiveness outcome	22 (4.9)	52 (3.5)	0.201
Primary safety outcome	6 (1.3)	6 (0.4)	**0.030**
Ischemic event	19 (4.2)	45 (3.1)	0.239
TIA	13 (2.9)	25 (1.7)	
Ischemic stroke	6 (1.3)	20 (1.4)	
Early neurological deterioration	15 (4.6)	36 (3.5)	0.407
Hemorrhagic transformation	9 (1.9)	10 (0.7)	**0.007**
Asymptomatic	7 (1.5)	10 (0.7)	
Symptomatic	2 (0.4)	0 (0)	
Intracranial hemorrhage	0 (0.0)	3 (0.2)	0.336
Subarachnoid hemorrhage	1 (0.2)	1 (0.1)	0.378
Other intracranial hemorrhage	1 (0.2)	1 (0.1)	0.378
Myocardial infarction	1 (0.2)	2 (0.1)	0.689
Death	3 (0.6)	5 (0.3)	0.351
Vascular	1 (0.2)	2 (0.1)	
Non‐Vascular	2 (0.4)	3 (0.2)	
Severe bleeding	3 (0.7)	6 (0.4)	0.488
Moderate bleeding	3 (0.7)	0 (0.0)	**0.002**
Mild bleeding	15 (3.3)	25 (1.7)	**0.035**
Any bleeding	13 (3.5)	31 (2.1)	**0.004**
New hospitalization	21 (4.6)	41 (2.8)	**0.030**
mRS	1 (0–2)	0 (0–1)	**0.001**

*Note:* Statistical significance was set as a *p*‐value < 0.05 and marked in bold. Abbreviations: LAA, large‐artery atherosclerosis; mRS, modified Rankin scale; *N*, number; TIA, transient ischemic attack.

In terms of DAPT safety, patients with LAA had a significantly higher rate of primary safety outcome (6, 1.3%) compared with non‐LAA patients (6, 0.4%, *p* = 0.030) (Table 2). This result was mainly driven by the significantly higher rates of patients with LAA presenting hemorrhagic transformation (9, 1.9%), although mainly asymptomatic (7, 1.5%), and moderate bleeding (3, 0.7%) compared with non‐LAA (10, 0.7% hemorrhagic transformations, *p* = 0.007; 0, moderate bleeding, *p* = 0.002) (Table 2). Also, the rate of any bleeding and its cumulative risk over the follow‐up were significantly higher in patients with LAA than in those with non‐LAA (Figure 4). The multivariate analyses confirmed LAA as a factor associated with the risk of any bleeding (HR 2.21 95% CI 1.27–3.86, *p* = 0.005) along with older age (HR 1.03, 95% CI 1.00–1.06, *p* = 0.018) (Table S3). Conversely, DAPT start after 24 h from the index event was associated with a reduced occurrence of any bleeding (HR 0.39, 95% CI 0.19–0.81, *p* = 0.012) (Table S3).

Among patients with LAA, those with ipsilateral carotid extracranial stenosis < 50% and ≥ 70% exhibited comparable outcomes in terms of DAPT effectiveness, safety, as well as similar index event features (Tables S4 and S5). Likewise, patients with intracranial stenosis and those with extracranial stenosis had similar outcomes (Figure S1).

#### 3.2.2 DAPT Effectiveness and Safety in LAA and Non‐LAA According to Pre‐Specified Subgroups

**FIGURE 5:**
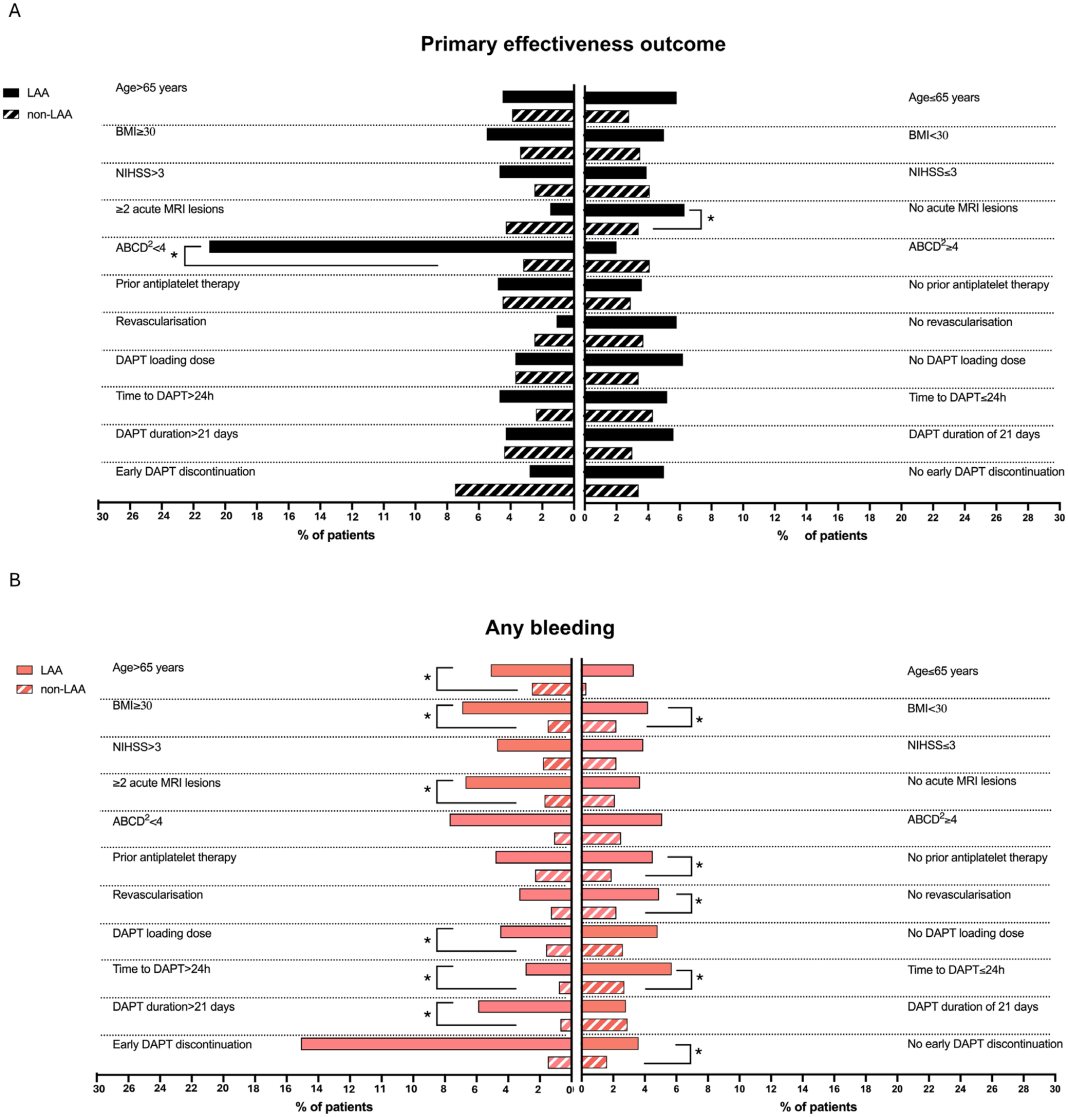
Comparison of primary effectiveness outcome (A) and any bleeding (B) between patients with large‐artery atherosclerosis and non‐large‐artery atherosclerosis across pre‐specified subgroups of interest. BMI, body mass index; DAPT, dual antiplatelet therapy; LAA, large‐artery atherosclerosis; NIHSS, National Institutes of Health Stroke Scale.

The subgroup analysis on the primary effectiveness outcomes showed a doubled rate of events in patients with LAA and a single MRI acute lesion compared with their counterparts (20, 6.3% vs. 42, 3.4%, respectively, *p* = 0.018). Similarly, the primary effectiveness outcome was significantly higher in patients with LAA and TIA reporting an ABCD^2^ score < 4 compared with those with non‐LAA (6, 23% vs. 3, 3.2%, respectively, *p* < 0.001). When considering the rate of any bleeding, patients with LAA had increased rates of events compared with those with non‐LAA. This increase was significantly different in those > 65 years (17, 5.1% vs. 24, 2.5%, *p* = 0.022), with ≥ 2 MRI acute lesions (9, 6.7% vs. 4, 1.7%; *p* = 0.014), those without prior antiplatelet treatment (12, 4.5% vs. 9, 2.5%; *p* = 0.020), and in those who did not undergo a revascularization procedure (18, 4.9% vs. 28, 2.2%, *p* = 0.007). Regarding the DAPT regimen, any bleeding was more frequent in patients with LAA who received a loading dose (11, 4.5% vs. 13, 1.6%, *p* = 0.009), those who received DAPT for more than 21 days (16, 5.9% vs. 4, 0.8%, *p* = 0.001), and those who did not discontinue the therapy early (15, 3.6% vs. 23, 1.6%, *p* = 0.013). Notably, the frequency of this outcome event was significantly higher in patients with LAA compared with those with non‐LAA independently from the BMI and time to DAPT start (Table S7 and Figure 5).

## 4 Discussion

The actual benefits and risks of DAPT in patients with LAA have not yet been fully established. The CLAIR and CARESS studies were the first to demonstrate the superiority of DAPT over SAPT in patients with LAA [22, 23]. However, these studies did not specifically include patients with minor ischemic stroke or high‐risk TIA, and their primary outcome was microembolism [22, 23]. RCTs involving patients with minor ischemic stroke and high‐risk TIA have shown heterogeneity in LAA definition and results [5, 6, 7, 8, 9]. This subgroup analysis of the READAPT study confirmed that the real‐world use of short‐term DAPT in patients with LAA differs from RCTs because of increased severity of the index event, delayed time to DAPT start, and prolonged treatment duration. In this study, we also observed that the vascular risk profile of patients with LAA was worse than the profile of those with non‐LAA. Rates of bleeding events were higher compared with RCTs [6, 7, 8, 9], despite similar or even lower rates of ischemic recurrences. At variance with RCTs, which excluded patients undergoing revascularization procedures and with moderate ischemic strokes [6, 7, 8, 9], we did not find a significantly increased risk of ischemic recurrences or a greater DAPT benefit in patients with LAA compared with those without. However, patients with LAA were exposed to a significantly higher bleeding risk.

We observed a 90‐day rate of ischemic recurrences in patients with LAA–4.2%—aligned with a wider population‐based study [3]. Ischemic recurrences were also within the range observed in RCTs—1.8%–10.6%—despite heterogeneity in definitions of LAA, minor stroke, and high‐risk TIA [6, 7, 8, 9] (Table 3). Similarly to most RCTs [6, 8, 9], we applied the TOAST classification for index event etiology [17]. However, still 30% of patients with ipsilateral extracranial carotid stenosis < 50% were classified as LAA by local investigators based on the characteristics of the atherothrombotic plaque and neuroimaging findings. Therefore, our cohort closely resembled that of the THALES trial, which included among the atherothrombotic events those occurring due to symptomatic 30%–50% extracranial carotid stenosis or ≥ 4 mm aortic plaque [7]. The lower rate of ischemic recurrences observed in READAPT compared with THALES subgroup analysis (7.2%) [7] requires cautious interpretation due to our study's observational design, which might have led to the underestimation of some outcomes events.

**TABLE 3:** Results of RCTs and READAPT on patients with an index event attributable to large‐artery atherosclerosis or to non‐large‐artery atherosclerosis.

	Ischemic recurrences, *N* (%)	ICH, *N* (%)	Any bleeding, *N* (%)
*READAPT*			
LAA (*N* = 451)	19 (4.2)	0 (0.0)	13 (3.5)
Non‐LAA (*N* = 1428)	52 (3.5)	3 (0.2)	31 (2.1)
*ATAMIS* [6]			
LAA (*N* = 112)	2 (1.8)	0 (0.0)	2 (1.7)
Non‐LAA (*N* = 1380)	10 (0.7)	2 (0.2)	8 (0.6)
*CHANCE‐2* [9]			
LAA (*N* = 825)	87 (10.6)^a^	1 (0.1)	19 (2.3)
SVO (*N* = 871)	58 (6.7)	3 (0.3)	19 (2.2)
SUE (*N* = 1478)	86 (5.8)	1 (0.1)	41 (2.8)
*THALES* [7]			
LAA (*N* = 2351)	87 (7.7)	4 (0.4)	N/A
Non‐LAA (*N* = 8665)	189 (4.3)	16 (0.4)	N/A

Abbreviations: ICH, intracranial hemorrhage; LAA, large‐artery atherosclerosis; *N*, number; N/A, not available; SUE, stroke of undetermined cause; SVO, small‐vessel occlusion.

^a^ Designated statistically significant ischemic risk when comparing LAA with non‐LAA.

Although the characteristics of the index event and DAPT regimen did not appear to influence ischemic recurrence risk, these factors, along with demographics, might partially explain the higher rate of bleeding events—3.2%—compared with RCTs [6, 7, 8, 9] (Table 3). Our cohort included older patients and higher rates of males than RCTs [10]—both factors were associated with an increased risk of any bleeding, as previously shown [24]. In our cohort, LAA was associated with an increased number of any bleedings, mainly driven by moderate bleedings and hemorrhagic transformations, while the rates of ICH and other intracerebral bleedings were null or similar to RCTs [6, 7, 8, 9]. The increased rate of moderate bleeding might be affected by prolonged DAPT duration, with around half of patients with LAA receiving DAPT for 30–90 days. Prolonged DAPT duration was a known risk factor for bleeding events [25] but still a treatment option in patients perceived at high risk for ischemic recurrences [13]. The high rate of hemorrhagic transformations can be, partly, attributed to the severity of the ischemic stroke—around 20% of patients had an NIHSS score > 5—possibly suggesting larger ischemic lesions, especially in those with anterior circulation and left hemisphere involvement [26]. An increased risk of cerebral amyloid angiopathy in patients with LAA is unlikely, as MRI signs of cSVD were similar to those observed in non‐LAA patients.

Similarly, when comparing patients with and without LAA, we observed higher rates of bleeding events and, specifically, hemorrhagic transformations in those with LAA compared with their counterparts. Additionally, patients with LAA had significantly longer DAPT duration, higher baseline NIHSS scores, and more frequently multiple acute lesions at MRI baseline scans compared with those with non‐LAA. Similar to subgroup analyses of the ATAMIS [6], THALES [7], and CHANCE‐2 [9] trials, we observed slightly higher rates of ischemic recurrences in patients with LAA compared to those with non‐LAA.

To identify patients most likely to benefit from DAPT, we compared primary effectiveness and any bleeding outcomes across subgroups of patients with LAA and those with non‐LAA. DAPT was less effective in reducing ischemic recurrences in patients with LAA and an ABCD^2^ score < 4 or in those with none or a single MRI acute lesion. However, patients with ≥ 2 MRI acute lesions had an increased risk of bleeding events, along with those aged ≥ 65 years. Among DAPT regimen characteristics, DAPT duration > 21 days was associated with higher rates of bleeding only in patients with LAA. Patients with LAA naïve to prior antiplatelet therapies and those receiving a loading dose had significantly higher bleeding events than their counterparts. Conversely, patients with LAA treated with revascularization procedures experienced similar bleeding events compared to those who did not, possibly because they did not receive a DAPT loading dose as previously discussed [10, 27]. The increased rate of bleeding in patients with LAA was independent from the time to DAPT start, despite a delayed time to DAPT start being safer in the entire study cohort and still beneficial as per RCTs data [8, 28]. Similarly, hemorrhagic risk in patients with LAA was independent from BMI cutoffs, suggesting no significant interaction between LAA and body weight. This supports previous findings that did not show an increased hemorrhagic risk related to BMI [29]. Overall, these findings underscore a hemorrhagic risk in patients with LAA likely tied to their overall vascular comorbidities.

Although READAPT had a large sample size, its open‐label observational design and limited ethnic diversity constrain the generalizability of our findings. We performed only exploratory analyses due to the low number of outcome events, possibly due to their underreporting [11]. Index event characteristics and outcome adjudication relied on local investigators, as we lacked direct access to patient records and neuroimaging, including scan timing. Without MRI scans, we could not consistently measure lesion volume or assess location and distribution. Moreover, some patients with non‐LAA might have had a significant atherosclerosis, though insufficient to classify as the cause of ischemic stroke or TIA according to the TOAST classification. Conversely, some patients with ipsilateral atherosclerosis may have been categorized as LAA without certainty of the causal role of atherosclerosis or of the absence of alternative causes. Variability in carotid stenosis assessments (e.g., epiaortic Doppler ultrasonography, CT angiography, or angiography) further limits our findings. Lastly, the single‐arm design of the study did not allow us to determine whether short‐term DAPT was superior to SAPT in patients with LAA.

In conclusion, this subgroup analysis of the READAPT study indicated a broader use of DAPT in LAA patients in the real world compared with RCTs settings. While patients with LAA face similar ischemic recurrence risks as non‐LAA patients in real‐world settings, they endure significantly higher bleeding risks. Therefore, short‐term DAPT should be carefully considered in LAA patients, particularly in older patients or those with more severe ischemic stroke (i.e., NIHSS > 3) and multiple acute lesions, as they may be at increased risk of intracranial bleeding. Our findings emphasize the need to tailor DAPT therapy according to the individual bleeding risk of LAA patients and the persistence of a knowledge gap from randomized studies in subgroups of patients at highest risk.

## Author Contributions

**Eleonora De Matteis:** conceptualization, writing – original draft, methodology, writing – review and editing, formal analysis, data curation, project administration. **Federico De Santis:** data curation, writing – review and editing. **Matteo Foschi:** data curation, writing – review and editing. **Michele Romoli:** data curation, writing – review and editing. **Tiziana Tassinari:** resources, writing – review and editing. **Valentina Saia:** resources, writing – review and editing. **Silvia Cenciarelli:** resources, writing – review and editing. **Chiara Bedetti:** resources, writing – review and editing. **Chiara Padiglioni:** resources, writing – review and editing. **Bruno Censori:** resources, writing – review and editing. **Valentina Puglisi:** resources, writing – review and editing. **Luisa Vinciguerra:** resources, writing – review and editing. **Maria Guarino:** resources, writing – review and editing. **Valentina Barone:** resources, writing – review and editing. **Marialuisa Zedde:** resources, writing – review and editing. **Ilaria Grisendi:** resources, writing – review and editing. **Ilaria Maestrini:** resources, writing – review and editing. **Maria Rosaria Bagnato:** resources, writing – review and editing. **Marco Petruzzellis:** resources, writing – review and editing. **Domenico Maria Mezzapesa:** resources, writing – review and editing. **Pietro Di Viesti:** resources, writing – review and editing. **Vincenzo Inchingolo:** resources, writing – review and editing. **Manuel Cappellari:** resources, writing – review and editing. **Mara Zenorini:** resources, writing – review and editing. **Paolo Candelaresi:** resources, writing – review and editing. **Vincenzo Andreone:** resources, writing – review and editing. **Giuseppe Rinaldi:** resources, writing – review and editing. **Alessandra Bavaro:** resources, writing – review and editing. **Anna Cavallini:** resources, writing – review and editing. **Stefan Moraru:** resources, writing – review and editing. **Maria Grazia Piscaglia:** resources, writing – review and editing. **Valeria Terruso:** resources, writing – review and editing. **Marina Mannino:** resources, writing – review and editing. **Alessandro Pezzini:** resources, writing – review and editing. **Giovanni Frisullo:** resources, writing – review and editing. **Francesco Muscia:** resources, writing – review and editing. **Maurizio Paciaroni:** resources, writing – review and editing. **Maria Giulia Mosconi:** resources, writing – review and editing. **Andrea Zini:** resources, writing – review and editing. **Ruggiero Leone:** resources, writing – review and editing. **Carmela Palmieri:** resources, writing – review and editing. **Letizia Maria Cupini:** resources, writing – review and editing. **Michela Marcon:** resources, writing – review and editing. **Rossana Tassi:** resources, writing – review and editing. **Enzo Sanzaro:** resources, writing – review and editing. **Cristina Paci:** resources, writing – review and editing. **Giovanna Viticchi:** resources, writing – review and editing. **Daniele Orsucci:** resources, writing – review and editing. **Anne Falcou:** resources, writing – review and editing. **Susanna Diamanti:** resources, writing – review and editing. **Roberto Tarletti:** resources, writing – review and editing. **Patrizia Nencini:** resources, writing – review and editing. **Eugenia Rota:** resources, writing – review and editing. **Federica Nicoletta Sepe:** resources, writing – review and editing. **Delfina Ferrandi:** resources, writing – review and editing. **Luigi Caputi:** resources, writing – review and editing. **Gino Volpi:** resources, writing – review and editing. **Salvatore La Spada:** resources, writing – review and editing. **Mario Beccia:** resources, writing – review and editing. **Claudia Rinaldi:** resources, writing – review and editing. **Vincenzo Mastrangelo:** resources, writing – review and editing. **Francesco Di Blasio:** writing – review and editing. **Paolo Invernizzi:** resources, writing – review and editing. **Giuseppe Pelliccioni:** resources, writing – review and editing. **Maria Vittoria De Angelis:** resources, writing – review and editing. **Laura Bonanni:** resources, writing – review and editing. **Giampietro Ruzza:** resources, writing – review and editing. **Emanuele Alessandro Caggia:** resources, writing – review and editing. **Monia Russo:** resources, writing – review and editing. **Agnese Tonon:** resources, writing – review and editing. **Maria Cristina Acciarri:** resources, writing – review and editing. **Sabrina Anticoli:** resources, writing – review and editing. **Cinzia Roberti:** resources, writing – review and editing. **Giovanni Manobianca:** resources, writing – review and editing. **Gaspare Scaglione:** resources, writing – review and editing. **Francesca Pistoia:** resources, writing – review and editing. **Alberto Fortini:** resources, writing – review and editing. **Antonella De Boni:** resources, writing – review and editing. **Alessandra Sanna:** resources, writing – review and editing. **Alberto Chiti:** resources, writing – review and editing. **Leonardo Barbarini:** resources, writing – review and editing. **Marcella Caggiula:** resources, writing – review and editing. **Maela Masato:** resources, writing – review and editing. **Massimo Del Sette:** resources, writing – review and editing. **Francesco Passarelli:** resources, writing – review and editing. **Maria Roberta Bongioanni:** resources, writing – review and editing. **Danilo Toni:** resources, supervision, conceptualization, writing – review and editing. **Stefano Ricci:** conceptualization, supervision, resources, writing – review and editing. **Simona Sacco:** supervision, resources, methodology, writing – review and editing, conceptualization, writing – original draft. **Raffaele Ornello:** conceptualization, writing – original draft, writing – review and editing, resources, supervision, methodology.

## Consent

The study was approved by the Internal Review Board of the University of L'Aquila (Italy) with the number 03/2021 and patients gave written informed consent according to the Declaration of Helsinki.

## Conflicts of Interest

A.Z. reports compensation from Angels Initiative, Boehringer‐Ingelheim, Daiichi Sankyo, CSL Behring, Bayer, and Astra Zeneca; and he is a member of ESO guidelines, ISA‐AII guidelines, and IRETAS steering committee. R.O. reports compensations from Novartis and Allergan, Teva Pharmaceutical Industries, Eli Lilly and Company. S.S. reports compensations from Novartis, NovoNordisk, Allergan, AstraZeneca, Pfizer Canada Inc., Eli Lilly and Company, Teva Pharmaceutical Industries, H. Lundbeck A/S, and Abbott Canada; employment by Università degli Studi dell'Aquila. M.Pa. reports compensation from Daiichi Sankyo Company, Bristol Myers Squibb, Bayer, and Pfizer Canada Inc. D.T. reports compensation from Alexion, AstraZeneca, Medtronic, and Pfizer. The other authors report no conflicts.

## Supporting information


Data S1.

## Data Availability

Data will be available upon request to the corresponding author.
