# Supplementary material for: Real‐World Dual Antiplatelet Therapy Use Exceeds Randomized Trials Boundaries With Possible Safety Issues in Patients With Large Artery Atherosclerosis—Insights From the READAPT Study
Source: Eur J Neurol. 2025 Apr 23;32(4):e70163. doi: 10.1111/ene.70163 (PMC12015746; doi:10.1111/ene.70163)
Supplement: Supplementary file 1 — Data S1. [file ENE-32-e70163-s001.docx]

## Supplementary

Figure S1: Comparison of the outcome events between patients with intracranial and extracranial carotid large-artery atherosclerosis. Abbreviations: large-artery atherosclerosis (LAA); non-significant (ns).

##

**
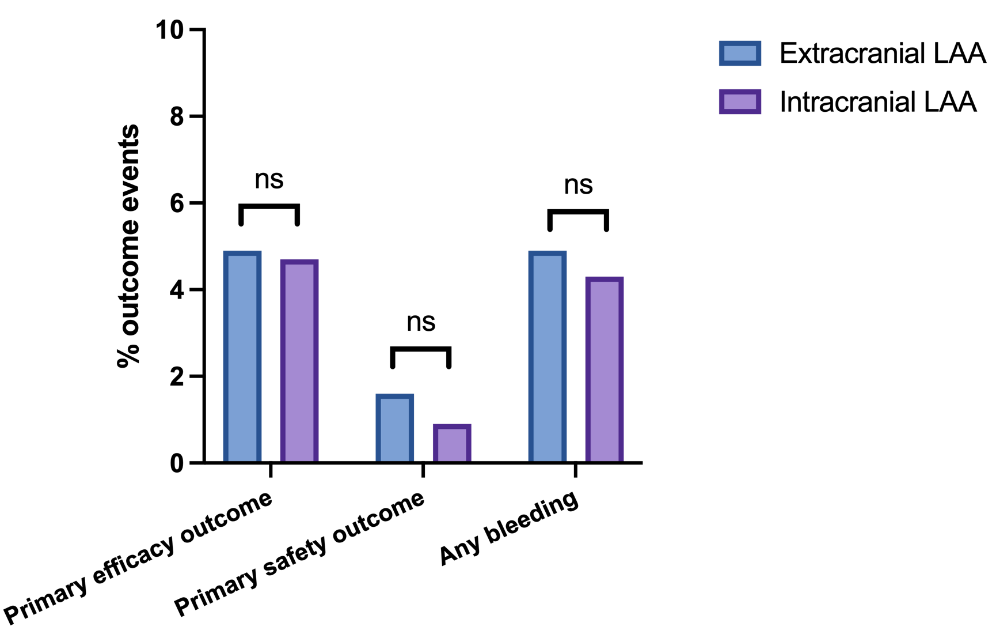
**

**Table S1**: Baseline MRI features of cerebral small vessel disease between patients with and without large-artery atherosclerosis

| **Covariates** | **Patients with LAA (N=319)** | **Non-LAA (N=997)** | **P value** |
| --- | --- | --- | --- |
| **White matter hyperintensities, N of patients (%)** | 210 (65.8) | 637 (63,9) | 0.529 |
| **Prior lacunar infarcts, N of patients (%)** | 151 (47.3) | 520 (52.2) | 0.134 |
| **Perivascular space, N of patients (%)** | 48 (15.0) | 182 (18.3) | 0.189 |
| **Cortical superficial siderosis, N of patients (%)** | 5 (1.1) | 12 (1.2) | 0.616 |
| **Microbleeds, N of patients (%)** | 12 (2.7) | 58 (5.8) | 0.154 |
| **Atrophy, N of patients (%)** | 56 (17.6) | 190 (12.9) | 0.549 |

Abbreviations: number (N); large-artery atherosclerosis (LAA).

**Table S2**: Univariate Cox regression to identify factors associated with primary effectiveness outcome

| **Covariates** | **HR (95% CI)** | **P value** |
| --- | --- | --- |
| **LAA** | 1.39 (0.84-2.28) | 0.2 |
| **Age** | 1.00 (0.98-0.1.02) | 0.9 |
| **Female gender** | 0.56 (0.32-0.96) | **0.03** |
| **Diabetes** | 1.21 (0.73-2.00) | 0.4 |
| **Hypercholesterolemia** | 0.72 (0.46-1.15) | 0.2 |
| **Hypertriglyceridemia** | 1.14 (0.67-1.97) | 0.6 |
| **Prior treatment with ASA** | 1.13 (0.70-1.82) | 0.6 |
| **Symptom duration** | 1.26 (0.78-2.03) | 0.3 |
| **Time to DAPT start >24h** | 0.70 (0.42-1.17) | 0.2 |
| **Early DAPT discontinuation** | 1.53 (0.61-3.80) | 0.4 |
| **DAPT duration >21 days** | 1.29 (0.82-2.04) | 0.3 |
| **IVT** | 0.53 (0.25-1.17) | 0.09 |

Abbreviations: aspirin (ASA); confidence interval (CI) dual antiplatelet therapy (DAPT); hazard ratio (HR); hours (h); intravenous thrombolysis (IVT); large-artery atherosclerosis (LAA).

**Table S3**: Univariate and Multivariate Cox regression to identify factors associated with any bleeding

|  | **Univariate** | | **Multivariate** | |
| --- | --- | --- | --- | --- |
| **Covariates** | **HR (95% CI)** | **P value** | **HR (95% CI)** | **P value** |
| **LAA** | 2.28 (1.28-3.87) | **0.006** | 2.21 (1.27-3.86) | **0.005** |
| **Age** | 1.03 (1.00-1.06) | **0.006** | 1.03 (1.00-1.06) | **0.018** |
| **Female gender** | 0.83 (0.46-1.50) | 0.5 | - | - |
| **Diabetes** | 1.09 (0.59-1.98) | 0.8 | - | - |
| **Hypercholesterolemia** | 1.04 (0.59-1.82) | 0.9 | - | - |
| **Hypertriglyceridemia** | 1.28 (0.68-2.39) | 0.5 | - | - |
| **Prior treatment with ASA** | 1.04 (0.59-1.84) | 0.9 | - | - |
| **Symptom duration** | 1.13 (0.63-2.01) | 0.7 | - | - |
| **Time to DAPT start >24h** | 0.39 (0.19-0.81) | **0.006** | 0.39 (0.19-0.81) | **0.012** |
| **DAPT duration >21 days** | 0.84 (0.48-1.47) | 0.5 | - | - |
| **Loading dose** | 0.73 (0.42-1.26) | 0.3 | - | - |
| **IVT** | 0.67 (0.28-1.56) | 0.3 | - | - |

Abbreviations: aspirin (ASA); confidence interval (CI) dual antiplatelet therapy (DAPT); hazard ratio (HR); hours (h); intravenous thrombolysis (IVT); large-artery atherosclerosis (LAA).

**Table S4**: Demographics and characteristics of the index event among patients with large-artery atherosclerosis index event and ipsilateral extracranial carotid stenosis 50-70% and >70%

| **Characteristics** | **Patients with LAA and <50% ipsilateral extracranial stenosis (N=55)** | **Patients with LAA and >70% ipsilateral extracranial stenosis (N=76)** | **P value** |
| --- | --- | --- | --- |
| **Age, years, median (IQR)** | 74 (69-81) | 72 (63-81) | 0.506 |
| **Female gender, N (%)** | 18 (32.7) | 22 (28.9) | 0.643 |
| **Caucasian, N (%)** | 55 (100) | 76 (100) | - |
| **BMI, median (IQR)** | 26 (24-28) | 25 (24-28) | 0.577 |
| **Current smoker, N (%)** | 12 (21.8) | 35 (46.1) | 0.098 |
| **Hypertension, N (%)** | 50 (90.9) | 66 (86.8) | 0.471 |
| **Diabetes mellitus, N (%)** | 25 (45.5) | 18 (23.7) | 0.009 |
| **Dyslipidemia, N (%)** | 37 (67.3) | 51 (67.1) | 0.984 |
| **Hypertriglyceridemia** | 10 (18.2) | 21 (27.6) | 0.209 |
| **Previous ischemic event (TIA or ischemic stroke), N (%)** | 13 (23.6) | 15 (19.7) | 0.591 |
| **Previous intracerebral hemorrhage, N (%)** | 0 (0.0) | 0 (0.0) | - |
| **Myocardial infarction, N (%)** | 4 (7.3) | 11 (14.8) | 0.201 |
| **Angina, N (%)** | 1 (1.8) | 3 (3.9) | 0.485 |
| **Congestive heart failure, N (%)** | 2 (3-6) | 2 (2.6) | 0.741 |
| **Peripheral Chronic Obliterative Arteriopathy, N (%)** | 2 (3.6) | 11 (14.5) | **0.041** |
| **Use of antiplatelet prior to the index event, N (%)** | 25 (45.5) | 29 (38.2) | 0.402 |
| **Symptom duration, N (%)**  **>24 h**  **<24 h** | 51 (92.7)  4 (7.3) | 71 (93.4)  5 (6.6) | 0.877 |
| **Lesions at neuroimaging, N (%)**  **Yes**  **No** | 52 (94.5)  3 (5.5) | 68 (89.5)  8 (10.5) | 0.302 |
| **ABCD^2^ score in patients with qualifying TIA, median (IQR)**  **ABCD2 <4, N (%)** | 4 (3-6)  1 (25.0) | 5 (4-6)  0 (0.0) | 0.556  0.236 |
| **NIHSS score in patients with qualifying ischemic stroke, median (IQR) and [range]**  **NIHSS>3, N (%)**  **NIHSS>5, N (%)** | 3 (2-5)  21 (41.2)  9 (17.6) | 3 (2-6)  35 (49.3)  24 (33.8) | 0.263  0.375  0.048 |
| **mRS baseline, median (IQR)** | 0 (0-1) | 0 (0-1) | 0.969 |
| **Time to DAPT start, N (%)**  **<12 h**  **12-24 h**  **25-48 h**  **>48 h** | 12 (21.8)  15 (27.3)  12 (21.8)  16 (29.1) | 26 (34.2)  19 (25.0)  14 (18.4)  17 (22.4) | 0.473 |
| **Type of DAPT, N (%)**  **Aspirin/Clopidogrel**  **Aspirin/Ticagrelor** | 53 (96.4)  2 (3.6) | 76 (100)  0 (0.0) | 0.094 |
| **Loading dose, N (%)**  **Aspirin**  **Clopidogrel**  **Ticagrelor** | 17 (30.9)  21 (38.2)  1 (1.8) | 46 (60.5)  28 (36.8)  29 (38.2)  0 (0.0) | 0.373  0.480  0.998  - |
| **Revascularization procedures, N (%)** | 11 (20.0) | 20 (26.3) | 0.401 |
| **DAPT duration, median (IQR)**  **DAPT duration <21 days, N (%)**  **DAPT duration 21-30 days, N (%)**  **DAPT duration 30-90 days, N (%)** | 30 (21-90)  1 (1.8)  32 (58.2)  22 (40.0) | 30 (21-90)  11 (14.5)  28 (36.8)  37 (48.7) | **0.010** |
| **DAPT discontinuation before expected completion, N (%)**  **Adverse events**  **Lack of compliance**  **Other** | 0 (0.0)  0 (0.0)  2 (3.6) | 2 (2.6)  0 (0.0)  8 (10.5) | 0.488 |

Abbreviations: Body mass index (BMI); Dual Antiplatelet Therapy (DAPT); Hours (h); Interquartile range (IQR); Loading dose (LD); large-artery atherosclerosis (LAA); modified Rankin scale (mRS); Number (N), National Institutes of Health Stroke Scale (NIHSS); transient ischemic attack (TIA). *all patients were evaluable for the analysis

**Table S5:** Outcomes among patients with large-artery atherosclerosis index event ipsilateral extracranial carotid stenosis <50% and >70%

| **Outcome** | **LAA patients with <50% ipsilateral extracranial stenosis (N=55)** | **LAA patients with >70% ipsilateral extracranial stenosis (N=76)** | **P value** |
| --- | --- | --- | --- |
| **Primary effectiveness outcome** | 2 (3.6) | 1 (1.3) | 0.393 |
| **Primary safety outcome** | 0 (0.0) | 1 (1.3) | 0.381 |
| **Ischemic event**  **Ischemic stroke  TIA** | 2 (3.6)  1 (1.8)  1 1(1.8) | 1 (1.3)  0 (0.0)  1 (1.3) | 0.381  0.386 |
| **Early neurological deterioration** | 3 (5.9) | 3 (3.9) | 0.676 |
| **Hemorrhagic transformation**  **Symptomatic**  **Asymptomatic** | 0 (0.0)  0 (0.0)  0 (0.0) | 3 (3.9)  2 (2.6)  1 (1.3) | 0.520 |
| **Intracranial hemorrhage** | 0 (0.0) | 0 (0.0) | - |
| **Subarachnoid hemorrhage** | 0 (0.0) | 0 (0.0) | - |
| **Other intracranial hemorrhage** | 0 (0.0) | 0 (0.0) | - |
| **Myocardial infarction** | 0 (0.0) | 0 (0.0) | - |
| **Death**  **Vascular**  **Non-Vascular** | 1 (1.8)  0 (0.0)  1 (1.8) | 0 (0.0)  0 (0.0)  0 (0.0) | 0.238  -  0.238 |
| **Severe bleeding** | 0 (0.0) | 0 (0.0) | - |
| **Moderate bleeding** | 0 (0.0) | 1 (1.3) | 0.393 |
| **Mild bleeding** | 2 (3.6) | 3 (3.9) | 0.927 |
| **Any bleeding** | 2 (3.6) | 4 (5.3) | 0.660 |
| **New hospitalization** | 3 (5.5) | 8 (10.5) | 0.302 |
| **mRS** | 1 (0-2) | 1 (0-2) | 0.450 |

Abbreviations: large-artery atherosclerosis (LAA); modified Rankin scale (mRS); Number (N), transient ischemic attack (TIA)

**Table S6**: Comparison of primary effectiveness outcome and any bleeding between patients with large-artery atherosclerosis and non- large-artery atherosclerosis across pre-specified subgroups of interests

|  | **Primary effectiveness outcome (N=74)** | | | **Any bleeding (N=52)** | | |
| --- | --- | --- | --- | --- | --- | --- |
| **Subgroups** | **LAA**  **N (%)** | **Non-LAA**  **N (%)** | **P value** | **LAA**  **N (%)** | **Non-LAA**  **N (%)** | **P value** |
| **Age**  **Age >65 years**  **Age ≤65 years** | 15/332 (4.5)  7/120 (5.8) | 37/944 (3.9)  15/524 (2.8) | 0.635  0.106 | 17/332 (5.1)  4/120 (3.3) | 24/944 (2.5)  7/524 (1.3) | **0.022**  0.128 |
| **BMI**  **BMI≥30**  **BMI<30** | 4/72 (5.5)  18/362 (5.0) | 7/204 (3.4)  45/1264 (3.5) | 0.428  0.295 | 5/72 (6.9)  16/380 (4.2) | 3/204 (1.5)  28/1264 (2.2) | **0.017**  **0.035** |
| **NIHSS**  **NIHSS >3**  **NIHSS ≤3** | 7/150 (4.7)  7/179 (3.9) | 8/325 (2.5)  26/688 (4.1) | 0.201  0.935 | 7/150 (4.7)  7/179 (3.9) | 6/325 (1.8)  15/688 (2.2) | 0.080  0.190 |
| **≥2 acute MRI lesions**  **Yes**  **No** | 2/134 (1.5)  20/318 (6.3) | 10/229 (4.3)  42/1239 (3.4) | 0.139  **0.018** | 9/134 (6.7)  12/318 (3.7) | 4/229 (1.7)  27/1239 (2.1) | **0.014**  0.105 |
| **ABCD^2^**  **ABCD^2^ <4**  **ABCD^2^ ≥4** | 6/26 (23.0)  2/97 (2.0) | 3/94 (3.2)  15/361 (4.1) | **0.001**  0.333 | 2/26 (7.7)  5/97 (5.1) | 1/94 (1.0)  9/361 (2.5) | 0.055  0.176 |
| **Prior antiplatelet therapy**  **Yes**  **No** | 9/187 (4.8)  13/265 (3.6) | 27/596 (4.5)  25/872 (2.9) | 0.872  0.106 | 9/187 (4.8)  12/265 (4.5) | 14/596 (2.3)  17/872 (1.9) | 0.080  **0.020** |
| **Revascularization procedure**  **Yes**  **No** | 1/89 (1.1)  21/363 (5.8) | 6/238 (2.5)  46/1230 (3.7) | 0.437  0.088 | 3/89 (3.3)  18/363 (4.9) | 3/238 (1.3)  28/1230 (2.2) | 0.206  **0.007** |
| **DAPT loading dose**  **Yes**  **No** | 9/244 (3.7)  13/208 (6.2) | 29/791 (3.7)  23/677 (3.4) | 0.987  0.069 | 11/244 (4.5)  10/208 (4.8) | 13/791 (1.6)  18/677 (2.6) | **0.009**  0.121 |
| **Time to DAPT>24 h**  **Yes**  **No** | 8/171 (4.7)  14/267 (5.2) | 12/488 (2.4)  40/980 (4.3) | 0.145  0.511 | 5/171 (2.9)  16/281 (5.7) | 4/488 (0.8)  27/980 (2.7) | **0.041**  **0.017** |
| **DAPT duration >21 days**  **Yes**  **No** | 12/273 (4.3)  10/179 (5.6) | 24/541 (4.4)  28/927 (3.0) | 0.979  0.084 | 16/273 (5.9)  5/179 (2.8) | 4/541 (0.7)  27/927 (2.9) | **0.001**  0.931 |
| **Early DAPT discontinuation**  **Yes**  **No** | 1/35 (2.8)  21/417 (5.0) | 4/53 (7.5)  48/1415 (3.4) | 0.352  0.121 | 6/35 (17.1)  15/417 (3.6) | 8/53 (1.5)  23/1415 (1.6) | 0.797  **0.013** |

Abbreviations: body mass index (BMI); dual antiplatelet therapy (DAPT); large-artery atherosclerosis (LAA); Number (N); National Institutes of Health Stroke Scale (NIHSS). Data were available for the entire study cohort.

**Table S7**: Comparison of primary effectiveness outcome and any bleeding between patients with large-artery atherosclerosis and non- large-artery atherosclerosis across pre-specified subgroups of interests

|  | **Primary effectiveness outcome (N=74)** | | | **Any bleeding (N=52)** | | |
| --- | --- | --- | --- | --- | --- | --- |
| **Subgroups** | **LAA**  **N (%)** | **Non-LAA**  **N (%)** | **P value** | **LAA**  **N (%)** | **Non-LAA**  **N (%)** | **P value** |
| **Age**  **Age >65 years**  **Age ≤65 years** | 15/332 (4.5)  7/120 (5.8) | 37/944 (3.9)  15/524 (2.8) | 0.635  0.106 | 17/332 (5.1)  4/120 (3.3) | 24/944 (2.5)  7/524 (1.3) | **0.022**  0.128 |
| **BMI**  **BMI≥30**  **BMI<30** | 4/72 (5.5)  18/362 (5.0) | 7/204 (3.4)  45/1264 (3.5) | 0.428  0.295 | 5/72 (6.9)  16/380 (4.2) | 3/204 (1.5)  28/1264 (2.2) | **0.017**  **0.035** |
| **NIHSS**  **NIHSS >3**  **NIHSS ≤3** | 7/150 (4.7)  7/179 (3.9) | 8/325 (2.5)  26/688 (4.1) | 0.201  0.935 | 7/150 (4.7)  7/179 (3.9) | 6/325 (1.8)  15/688 (2.2) | 0.080  0.190 |
| **≥2 acute MRI lesions**  **Yes**  **No** | 2/134 (1.5)  20/318 (6.3) | 10/229 (4.3)  42/1239 (3.4) | 0.139  **0.018** | 9/134 (6.7)  12/318 (3.7) | 4/229 (1.7)  27/1239 (2.1) | **0.014**  0.105 |
| **ABCD^2^**  **ABCD^2^ <4**  **ABCD^2^ ≥4** | 6/26 (23.0)  2/97 (2.0) | 3/94 (3.2)  15/361 (4.1) | **0.001**  0.333 | 2/26 (7.7)  5/97 (5.1) | 1/94 (1.0)  9/361 (2.5) | 0.055  0.176 |
| **Prior antiplatelet therapy**  **Yes**  **No** | 9/187 (4.8)  13/265 (3.6) | 27/596 (4.5)  25/872 (2.9) | 0.872  0.106 | 9/187 (4.8)  12/265 (4.5) | 14/596 (2.3)  17/872 (1.9) | 0.080  **0.020** |
| **Revascularization procedure**  **Yes**  **No** | 1/89 (1.1)  21/363 (5.8) | 6/238 (2.5)  46/1230 (3.7) | 0.437  0.088 | 3/89 (3.3)  18/363 (4.9) | 3/238 (1.3)  28/1230 (2.2) | 0.206  **0.007** |
| **DAPT loading dose**  **Yes**  **No** | 9/244 (3.7)  13/208 (6.2) | 29/791 (3.7)  23/677 (3.4) | 0.987  0.069 | 11/244 (4.5)  10/208 (4.8) | 13/791 (1.6)  18/677 (2.6) | **0.009**  0.121 |
| **Time to DAPT>24 h**  **Yes**  **No** | 8/171 (4.7)  14/267 (5.2) | 12/488 (2.4)  40/980 (4.3) | 0.145  0.511 | 5/171 (2.9)  16/281 (5.7) | 4/488 (0.8)  27/980 (2.7) | **0.041**  **0.017** |
| **DAPT duration >21 days**  **Yes**  **No** | 12/273 (4.3)  10/179 (5.6) | 24/541 (4.4)  28/927 (3.0) | 0.979  0.084 | 16/273 (5.9)  5/179 (2.8) | 4/541 (0.7)  27/927 (2.9) | **0.001**  0.931 |
| **Early DAPT discontinuation**  **Yes**  **No** | 1/35 (2.8)  21/417 (5.0) | 4/53 (7.5)  48/1415 (3.4) | 0.352  0.121 | 6/35 (17.1)  15/417 (3.6) | 8/53 (1.5)  23/1415 (1.6) | 0.797  **0.013** |

Abbreviations: body mass index (BMI); dual antiplatelet therapy (DAPT); large-artery atherosclerosis (LAA); Number (N); National Institutes of Health Stroke Scale (NIHSS). Data were available for the entire study cohort.

**Table S8:** List of participating centres

| City | Site | PI | Co-investigator |
| --- | --- | --- | --- |
| Alessandria | Stroke Unit-Department of Neurology, SS. Biagio e Arrigo | Federica Nicoletta Sepe  Delfina Ferrandi | Martina Valente |
| Ancona | Experimental Medicine and clinical Department-Marche Polytechnic University | Giovanna Viticchi | Silvia Paolucci |
| Ancona | Department of Neurology INRCA | Giuseppe Pelliccioni | Leonardo Biscetti; Valentina Cameriere; Eleonora Potente |
| Acquaviva delle Fonti | Department of Neurology and Stroke Unit, Ente Ecclesiastico ospedale Generale Regionale Miulli | Giovanni Manobianca; Gaspare Scaglione |  |
| Avezzano | Department of Biotechnological and Applied Clinical Sciences-University of L'Aquila-SS Filippo e Nicola Hospital | Simona Sacco | Eleonora De Matteis;  Raffaele Ornello; Federico De Santis |
| Bari | Department of Neurology-Di Venere Hospital | Giuseppe Rinaldi | Alessandra Bavaro |
| Bari | Department of Neurology and Stroke Unit- "F. Puca" AOU Consorziale Policlinico | Marco Petruzzellis | Domenico Maria Mezzapesa; Martina Caccamo; Debora Galotto |
| Barletta | Department of Neurology and Stroke Unit, "M. R. Dimiccoli" General Hospital, Barletta, ASL BT, Italy | Ruggiero Leone | Sergio Altomare, Maurizio Giorelli |
| Bologna | IRCCS Istituto delle Scienze Neurologiche di Bologna, Bologna-Policlinico S.Orsola-Malpighi | Maria Guarino | Valentina Barone |
| Bologna | IRCCS Istituto delle Scienze Neurologiche di Bologna, Department of Neurology and Stroke Center-Maggiore Hospital | Andrea Zini | Federica Naldi; Chiara Bassi |
| Brescia | Department of Neurology, Istituto Ospedaliero Fondazione Poliambulanza | Paolo Invernizzi |  |
| Brindisi | Department of Neurology- Antonio Perrino Hospital | Salvatore La Spada |  |
| Cesena | Department of Neurology and Stroke Unit-Bufalini Hospital | Michele Romoli | Claudia Faini |
| Chieti | Clinics of Neurology, Stroke Unit, ospedale SS Annunziata, Stroke Unit Ospedale SS Annunziata | Maria Vittoria De Angelis; Laura Bonanni | Paola Ajdinaj; Anna Di Giovanni |
| Città di Castello | Department of Neurology-Città di Castello Hospital | Silvia Cenciarelli | Chiara Bedetti; Chiara Padiglioni; Elisa Sacchini |
| Cittadella | Department of Medicine-Cittadella Hospital | Giampietro Ruzza |  |
| Crema | Department of Cardiocerebrovascular diseases; Neurology-Stroke Unit-ASST Ospedale Maggiore di Crema | Luigi Caputi | Antonio Zito |
| Cremona | Department of Neurology-ASST Cremona Hospital | Bruno Censori | Valentina Puglisi; Alessia Giossi, Luisa Vinciguerra |
| Fermo | Department of Neurology-Fermo Hospital | Maria Cristina Acciarri | Patrizio Cardinali; Stefania Martina Angelocola |
| Florence | Stroke Unit-Careggi University Hospital | Patrizia Nencini | Laura Tudisco |
| Florence | Internal Medicine, San Giovanni di Dio Hospital | Alberto Fortini | Maraia Cristina Baruffi; Chiara Alessi |
| Genoa | Neuroscience, IRCCS Ospedale Policlinico San Martino | Massimo Del Sette | Alessandro Canessa, Davide Sassos |
| L'Aquila | Department of Biotechnological and Applied Clinical Sciences-University of L'Aquila-San Salvatore Hospital | Francesca Pistoia | Maria Grazia Vittorini |
| Legnano | Department of Neurology - ASST-Ovest Milanese | Francesco Muscia | Serena Gallo Cassarino; Gloria Valcamonica |
| Lecce | Department of Neurology-Vito Fazi Hospital | Leonardo Barbarini;  Marcella Caggiula | Annalisa Rizzo |
| Lucca | Unit of Neurology-San Luca Hospital | Daniele Orsucci | Marco Vista |
| Massa Carrara | Unit of Neurology, Apuane Hospital | Alberto Chiti |  |
| Milan | Department of Neurology and Stroke Unit San Giuseppe Hospital | Maurizio Paciaroni |  |
| Mirano-Venice | Department of Neurology- ULSS 3 Serenissima | Maela Masato | Elisabetta Menegazzo |
| Monza | Department of Neurology, Fondazione IRCCS San Gerardo dei Tintori Monza | Simone Beretta | Susanna Diamanti; Carlo Ferrarese |
| Naples | Department of Neurology and Stroke Unit, AORN Antonio Cardarelli | Paolo Candelaresi | Vincenzo Andreone; Antonio De Mase; Emanuele Spina |
| Novara | SCDU Neurologia - Stroke Unit, Azienda Ospedaliero-Universitaria "Maggiore | Roberto Tarletti | Thomas Fleetwood |
| Novi Ligure | Department of Neurology-San Giacomo Hospital | Eugenia Rota | Gian Luca Bruzzone |
| Palermo | Department of Neurology, AOOR Villa Sofia-Cervello | Valeria Terruso | Marina Mannino |
| Parma | Department of Medicine and Surgery, University of Parma, Stroke Care Program, Department of Emergency, Parma University Hospital, | Alessandro Pezzini | Antonio Genovese |
| Pavia | UO Neurologia d'Urgenza e Stroke Unit -IRCCS Mondino Foundation | Anna Cavallini | Stefan Moraru |
| Perugia | Internal and Cardiovascular Medicine - University of Perugia - Santa Maria della Misericordia Hospital | Maurizio Paciaroni  Maria Giulia Mosconi |  |
| Pescara | Stroke Unit -"S.Spirito" Hospital | Francesco Di Blasio | Daniela Monaco; Pierluigi Tocco; Maria Vittoria De Angelis |
| Pietra Ligure | Department of Neurology, Santa Corona Hospital | Tiziana Tassinari | Valentina Saia |
| Pinerolo | Department of Neurology, Stroke Unit, E. Agnelli Hospital, Pinerolo, Italy | Carmela Palmieri |  |
| Pistoia | Department of Neurology-San Jacopo Hospital | Gino Volpi | Chiara Menichetti; Federica Letteri; Serena Nannucci |
| Ragusa | Cardio-neuro-vascular Department-Neurology Unit -Giovanni Paolo II Hospital | Emanuele Alessandro Caggia |  |
| Ravenna | Department of Neuroscience-S.Maria delle Croci Hospital, AUSL Romagna, Ravenna | Matteo Foschi | Pietro Querzani |
| Reggio Emilia | Department of Neurology, AUSL-IRCCS di Reggio Emilia | Marialuisa Zedde | Ilaria Grisendi, Rosario Pascarella, Federica Assenza |
| Rimini | Neurology Unit, "Infermi" Hospital, AUSL Romagna | Claudia Rinaldi,  Vincenzo Mastrangelo | Enrico Maria Lotti  Giorgia Bernabè |
| Rome | Department of Neurology -Sant'Andrea Hospital | Mario Beccia | Filomena Di Lisi |
| Rome | Department of Systems Medicine-Tor Vergata University Hospital | Marina Diomedi | Maria Rosaria Bagnato; Ilaria Maestrini |
| Rome | Department of Neurology and Stroke Unit- S. Eugenio Hospital | Letizia Maria Cupini | Novella Bonaffini; Maria Chiara Ricciardi |
| Rome | Stroke Unit, Azienda Ospedaliera San Camillo, | Sabrina Anticoli | Chiara De Fino |
| Rome | Department of Neurology-San Filippo Neri Hospital | Cinzia Roberti |  |
| Rome | Department of Neurology- Fatebenefratelli Hospital | Francesco Passarelli |  |
| Rome | Stroke Unit, Emergency Department, Policlinico Umberto I Hospital, Rome, Italy | Anna Falcou | Nicoletta Giuseppa Caracciolo, Manuela De Michele |
| Rome | Neuroscienze, Organi di Senso e Torace, Fondazione Policlinico Universitario Agostino Gemelli | Giovanni Frisullo | Irene Scala; Simone Bellavia |
| Rovigo | Department of Neurology-SM Misericordia Hospital | Monia Russo |  |
| San Benedetto del Tronto | UOC Neurologia, Ospedale "Madonna del Soccorso" | Cristina Paci | Giulio Papiri; Emanuele Puca |
| San Giovanni Rotondo | Department of Neurology Casa sollievo della sofferenza | Pietro Di Viesti; Vincenzo Inchingolo | Giovanni Matteo Fratta |
| Sassari | Stroke Unit-AOU Sassari | Alessandra Sanna |  |
| Savigliano | Department of Neurology-SS Annunziata Hospital | Maria Roberta Bongioanni |  |
| Siena | Urgency and Emergency Department -Azienda Ospedaliera Universitaria Senese | Rossana Tassi | Ivo Giuseppe De Franco; Maurizio Acampa |
| Siracusa | Department of Neurology, Umberto I Hospital, | Enzo Sanzaro | Roberto Cappellani; Eleonora Sgarlata |
| Venice | Department of Neurology, Ospedale Civile SS. Giovanni e Paolo, Venezia, Italy | Agnese Tonon |  |
| Verona | Department of Neuroscience-Azienda Ospedaliera Universitaria Intergrata Verona | Manuel Cappellari | Mara Zenorini; Cecilia Zivelonghi |
| Vicenza | Department of Neuroscience-San Bortolo Hospital | Antonella De Boni | Cristina De Luca; Francesco Perini |
| Vicenza | Department of Neurology, Cazzavillan Hospital Arzignano, Vicenza, Italy | Michela Marcon |  |

##

## READAPT group authorship

Maurizio Acampa, Urgency and Emergency Department -Azienda Ospedaliera Universitaria Senese, Siena, Italy

Maria Cristina Acciarri, Department of Neurology-Fermo Hospital, Fermo, Italy

Paola Ajdinaj, Clinics of Neurology, Stroke Unit, ospedale SS Annunziata, Stroke Unit Ospedale SS Annunziata, Chieti, Italy

Sergio Altomare, Department of Neurology and Stroke Unit, "M. R. Dimiccoli" General Hospital, Barletta, ASL BT, Italy

Chiara Alessi, Internal Medicine, San Giovanni di Dio Hospital, Florence, Italy

Vincenzo Andreone, Department of Neurology and Stroke Unit, AORN Antonio Cardarelli, Naples, Italy

Stefania Martina Angelocola, Department of Neurology-Fermo Hospital, Fermo, Italy

Sabrina Anticoli, Stroke Unit, Azienda Ospedaliera San Camillo, Rome, Italy

Federica Assenza, Neurology Unit, Stroke Unit, Azienda Unità Sanitaria Locale-IRCCS di Reggio Emilia, Reggio Emilia

Maria Rosaria Bagnato, Department of Systems Medicine-Tor Vergata University Hospital, Rome, Italy

Leonardo Barbarini, Department of Neurology-Vito Fazi Hospital, Lecce, Italy

Chiara Bassi, IRCCS Istituto delle Scienze Neurologiche di Bologna, Department of Neurology and Stroke Center-Maggiore Hospital, Bologna, Italy

Valentina Barone, IRCCS Istituto delle Scienze Neurologiche di Bologna, Bologna-Policlinico S.Orsola-Malpighi, Bologna, Italy

Maraia Cristina Baruffi, Internal Medicine, San Giovanni di Dio Hospital, Florence, Italy

Alessandra Bavaro, Department of Neurology, Di Venere Hospital, Bari, Italy

Mario Beccia, Department of Neurology -Sant'Andrea Hospital, Rome, Italy

Chiara Bedetti, Department of Neurology-Città di Castello Hospital, Città di Castello, Italy

Simone Bellavia, Neuroscienze, Organi di Senso e Torace, Fondazione Policlinico Universitario Agostino Gemelli, Rome, Italy

Simone Beretta, Department of Neurology, Fondazione IRCCS San Gerardo dei Tintori Monza, Monza, Italy

Giorgia Bernabè, Neurology Unit, "Infermi" Hospital, AUSL Romagna, Rimini, Italy

Leonardo Biscetti, Department of Neurology INRCA, Ancona, Italy

Novella Bonaffini, Department of Neurology and Stroke Unit- S. Eugenio Hospital, Rome, Italy

Laura Bonanni, Clinics of Neurology, Stroke Unit, ospedale SS Annunziata, Stroke Unit Ospedale SS Annunziata, Chieti, Italy

Maria Roberta Bongioanni, Department of Neurology-SS Annunziata Hospital, Savigliano, Italy

Gian Luca Bruzzone, Department of Neurology-San Giacomo Hospital, Novi Ligure, Italy

Martina Caccamo, Department of Neurology and Stroke Unit- "F. Puca" AOU Consorziale Policlinico, Bari, Italy

Marcella Caggiula, Department of Neurology, Vito Fazi Hospital, Lecce, Italy

Valentina Cameriere, Department of Neurology INRCA, Ancona, Italy

Paolo Candelaresi, Department of Neurology and Stroke Unit, AORN Antonio Cardarelli, Naples, Italy

Alessandro Canessa, Neuroscience, IRCCS Ospedale Policlinico San Martino, Genoa, Italy

Luigi Caputi, Department of Cardiocerebrovascular diseases; Neurology-Stroke Unit-ASST Ospedale Maggiore di Crema, Crema, Italy

Nicoletta Giuseppa Caracciolo, Department of Human neurosciences, University of Rome La Sapienza, Rome, Italy

Patrizio Cardinali, Department of Neurology-Fermo Hospital, Fermo, Italy

Anna Cavallini, UO Neurologia d'Urgenza e Stroke Unit -IRCCS Mondino Foundation, Pavia, Italy

Emanuele Alessandro Caggia, Cardio-neuro-vascular Department-Neurology Unit -Giovanni Paolo II Hospital, Ragusa, Italy

Roberto Cappellani, Department of Neurology, Umberto I Hospital, Siracusa, Italy

Manuel Cappellari, Department of Neuroscience-Azienda Ospedaliera Universitaria Intergrata Verona, Verona, Italy

Silvia Cenciarelli, Department of Neurology-Città di Castello Hospital, Città di Castello, Italy

Bruno Censori, Department of Neurology-ASST Cremona Hospital, Cremona, Italy

Alberto Chiti, Unit of Neurology, Apuane Hospital, Massa Carrara, Italy,

Letizia Maria Cupini, Department of Neurology and Stroke Unit- S. Eugenio Hospital, Rome, Italy

Maria Vittoria De Angelis, Department of Neurology and Stroke Unit, SS Annunziata Hospital, Chieti, Italy, Chieti, Italy and Stroke Unit -"S.Spirito" Hospital, Pescara, Italy

Antonella De Boni, Department of Neuroscience-San Bortolo Hospital, Vicenza, Italy

Chiara De Fino, Stroke Unit, Azienda Ospedaliera San Camillo, Rome, Italy

Cristina De Luca, Department of Neuroscience-San Bortolo Hospital, Vicenza, Italy

Antonio De Mase, Department of Neurology and Stroke Unit, AORN Antonio Cardarelli, Naples, Italy

Eleonora De Matteis, Department of Biotechnological and Applied Clinical Sciences-University of L'Aquila-SS Filippo e Nicola Hospital, L’Aquila, Italy and Department of Brain Sciences, Faculty of Medicine, Imperial College London, London, UK

Manuela De Michele, Stroke Unit, Emergency Department, Policlinico Umberto I Hospital, Rome, Italy

Federico De Santis, Department of Biotechnological and Applied Clinical Sciences-University of L'Aquila-SS Filippo e Nicola Hospital, L’Aquila, Italy

Massimo Del Sette, Neuroscience, IRCCS Ospedale Policlinico San Martino, Genoa, Italy

Francesco Di Blasio, Stroke Unit -"S.Spirito" Hospital, Pescara, Italy

Ivo Giuseppe De Franco, Urgency and Emergency Department -Azienda Ospedaliera Universitaria Senese, Siena, Italy

Anna Di Giovanni, Clinics of Neurology, Stroke Unit, ospedale SS Annunziata, Stroke Unit Ospedale SS Annunziata, Chieti, Italy

Filomena Di Lisi, Department of Neurology -Sant'Andrea Hospital, Rome, Italy

Pietro Di Viesti, Department of Neurology Casa sollievo della sofferenza, San Giovanni Rotondo, Italy

Susanna Diamanti, Department of Neurology, Fondazione IRCCS San Gerardo dei Tintori Monza, Monza, Italy

Marina Diomedi, Department of Systems Medicine-Tor Vergata University Hospital, Rome, Italy

Anna Falcou, Stroke Unit, Emergency Department, Policlinico Umberto I Hospital, Rome, Italy

Claudia Faini, Department of Neurology and Stroke Unit-Bufalini Hospital, Cesena, Italy

Ciro Alberto Fasolino, Department of Human Neurosciences, Interventional Neuroradiology and Neurology, University of Rome La Sapienza, Rome, Italy

Delfina Ferrandi, Stroke Unit-Department of Neurology, SS. Biagio e Arrigo, Alessandria. Italy

Carlo Ferrarese, Department of Neurology, Fondazione IRCCS San Gerardo dei Tintori Monza, Monza, Italy

Thomas Fleetwood, SCDU Neurologia - Stroke Unit, Azienda Ospedaliero-Universitaria "Maggiore, Novara, Italy

Alberto Fortini, Internal Medicine, San Giovanni di Dio Hospital, Florence, Italy

Matteo Foschi, Department of Biotechnological and Applied Clinical Sciences, University of L'Aquila, L’Aquila, Italy, and Department of Neuroscience-S.Maria delle Croci Hospital, AUSL Romagna, Ravenna, Italy

Giovanni Matteo Fratta, Department of Neurology Casa sollievo della sofferenza, San Giovanni Rotondo, Italy

Giovanni Frisullo, Neuroscienze, Organi di Senso e Torace, Fondazione Policlinico Universitario Agostino Gemelli, Rome, Italy

Antonio Genovese, Department of Medicine and Surgery, University of Parma, Stroke Care Program, Department of Emergency, Parma University Hospital, Parma, Italy

Serena Gallo Cassarino, Department of Neurology - ASST-Ovest Milanese, Legnano, Italy

Debora Galotto, Department of Neurology and Stroke Unit- "F. Puca" AOU Consorziale Policlinico, Bari, Italy

Maurizio Giorelli, Department of Neurology and Stroke Unit, "M. R. Dimiccoli" General Hospital, Barletta, ASL BT, Italy

Alessia Giossi, Department of Neurology-ASST Cremona Hospital, Cremona, Italy

Ilaria Grisendi, Neurology Unit, Stroke Unit, Azienda Unità Sanitaria Locale-IRCCS di Reggio Emilia, Reggio Emilia

Maria Guarino, IRCCS Istituto delle Scienze Neurologiche di Bologna, Bologna, Italy

Vincenzo Inchingolo, Department of Neurology Casa sollievo della sofferenza, San Giovanni Rotondo, Italy

Paolo Invernizzi, Department of Neurology, Istituto Ospedaliero Fondazione Poliambulanza, Brescia, Italy

Salvatore La Spada, Department of Neurology- Antonio Perrino Hospital. Brindisi, Italy

Ruggiero Leone, Operative Unit of Neurology, "Dimiccoli" General Hospital, Barletta, ASL BT, Italy.

Federica Letteri, Department of Neurology-San Jacopo Hospital, Pistoia, Italy

Enrico Maria Lotti, Neurology Unit, "Infermi" Hospital, AUSL Romagna, Rimini, Italy

Ilaria Maestrini, Department of Systems Medicine-Tor Vergata University Hospital, Rome, Italy

Marina Mannino, Department of Neurology, AOOR Villa Sofia-Cervello, Palermo, Italy

Giovanni Manobianca, Department of Neurology and Stroke Unit, Ente Ecclesiastico ospedale Generale Regionale Miulli, Acquaviva delle Fonti, Italy

Michela Marcon, Department of Neurology, Cazzavillan Hospital Arzignano, Vicenza, Italy

Maela Masato, Department of Neurology- ULSS 3 Serenissima, Mirano-Venice, Italy

Vincenzo Mastrangelo, Neurology Unit, "Infermi" Hospital, AUSL Romagna, Rimini, Italy

Chiara Menichetti, Department of Neurology-San Jacopo Hospital, Pistoia, Italy

Elisabetta Menegazzo, Department of Neurology- ULSS 3 Serenissima, Mirano-Venice, Italy

Domenico Maria Mezzapesa, Department of Neurology and Stroke Unit- "F. Puca" AOU Consorziale Policlinico, Bari, Italy

Daniela Monaco, Stroke Unit -"S.Spirito" Hospital, Pescara, Italy

Stefan Moraru, UO Neurologia d'Urgenza e Stroke Unit -IRCCS Mondino Foundation, Pavia, Italy

Maria Giulia Mosconi, Internal and Cardiovascular Medicine - University of Perugia - Santa Maria della Misericordia Hospital, Perugia, Italy

Francesco Muscia, Department of Neurology - ASST-Ovest Milanese, Legnano, Italy

Federica Naldi, IRCCS Istituto delle Scienze Neurologiche di Bologna, Department of Neurology and Stroke Center-Maggiore Hospital, Bologna, Italy

Serena Nannucci, Department of Neurology-San Jacopo Hospital, Pistoia, Italy

Patrizia Nencini, Stroke Unit-Careggi University Hospital, Florence, Italy

Raffaele Ornello, Department of Biotechnological and Applied Clinical Sciences-University of L'Aquila-SS Filippo e Nicola Hospital, L’Aquila, Italy

Daniele Orsucci, Unit of Neurology-San Luca Hospital, Lucca, Italy

Cristina Paci, UOC Neurologia, Ospedale "Madonna del Soccorso", San Benedetto del Tronto, Italy

Maurizio Paciaroni, Department of Neurosciences and Rehabilitation, University of Ferrara, Ferrara, Italy

Chiara Padiglioni, Department of Neurology-Città di Castello Hospital, Città di Castello, Italy

Carmela Palmieri, Department of Neurology, Stroke Unit, E. Agnelli Hospital, Pinerolo, Italy

Silvia Paolucci, Experimental Medicine and clinical Department-Marche Polytechnic University, Ancona, Italy

Giulio Papiri, UOC Neurologia, Ospedale "Madonna del Soccorso", San Benedetto del Tronto, Italy

Rosario Pascarella, Neurology Unit, Stroke Unit, Azienda Unità Sanitaria Locale-IRCCS di Reggio Emilia, Reggio Emilia

Francesco Passarelli, Department of Neurology- Fatebenefratelli Hospital, Rome, Italy

Giuseppe Pelliccioni, Department of Neurology INRCA, Ancona, Italy

Francesco Perini, Department of Neuroscience-San Bortolo Hospital, Vicenza, Italy

Marco Petruzzellis, Department of Neurology and Stroke Unit- "F. Puca" AOU Consorziale Policlinico, Bari, Italy

Francesca Pistoia, Department of Biotechnological and Applied Clinical Sciences-University of L'Aquila, L’Aquila, Italy

Eleonora Potente, Department of Neurology INRCA, Ancona, Italy

Emanuele Puca, UOC Neurologia, Ospedale "Madonna del Soccorso", San Benedetto del Tronto, Italy

Valentina Puglisi, Department of Neurology-ASST Cremona Hospital, Cremona, Italy

Pietro Querzani, Department of Neuroscience-S.Maria delle Croci Hospital, AUSL Romagna, Ravenna

Stefano Ricci, Department of Neurology, Città di Castello Hospital, Città di Castello, Italy and Coordinatore Comitato Scientifico ISA-AII

Maria Chiara Ricciardi, Department of Neurology and Stroke Unit- S. Eugenio Hospital, Rome, Italy

Claudia Rinaldi, Neurology Unit, "Infermi" Hospital, AUSL Romagna, Rimini, Italy

Giuseppe Rinaldi, Department of Neurology, Di Venere Hospital, Bari, Italy

Annalisa Rizzo, Department of Neurology, Vito Fazi Hospital, Lecce, Italy

Cinzia Roberti, Department of Neurology-San Filippo Neri Hospital, Rome, Italy

Michele Romoli, Department of Neurology and Stroke Unit-Bufalini Hospital, Cesena, Italy

Eugenia Rota, Department of Neurology-San Giacomo Hospital, Novi Ligure, Italy

Monia Russo, Department of Neurology-SM Misericordia Hospital, Rovigo, Italy

Giampietro Ruzza, Department of Medicine-Cittadella Hospital, Cittadella, Italy

Elisa Sacchini, Department of Neurology-Città di Castello Hospital, Città di Castello, Italy

Simona Sacco, Department of Biotechnological and Applied Clinical Sciences-University of L'Aquila-SS Filippo e Nicola Hospital, L’Aquila, Italy

Valentina Saia, Department of Neurology-Santa Corona Hospital, Pietra Ligure, Italy

Alessandra Sanna, Stroke Unit-AOU Sassari, Sassari, Italy

Enzo Sanzaro, Department of Neurology, Umberto I Hospital, Siracusa, Italy

Davide Sassos, Neuroscience, IRCCS Ospedale Policlinico San Martino, Genoa, Italy

Gaspare Scaglione, Department of Neurology and Stroke Unit, Ente Ecclesiastico ospedale Generale Regionale Miulli, Acquaviva delle Fonti, Italy

Irene Scala, Neuroscienze, Organi di Senso e Torace, Fondazione Policlinico Universitario Agostino Gemelli, Rome, Italy

Alessandro Pezzini, Department of Medicine and Surgery, University of Parma, Stroke Care Program, Department of Emergency, Parma University Hospital, Parma, Italy Federica Nicoletta Sepe, Stroke Unit-Department of Neurology, SS. Biagio e Arrigo, Alessandria. Italy

Eleonora Sgarlata, Department of Neurology, Umberto I Hospital, Siracusa, Italy

Emanuele Spina, Department of Neurology and Stroke Unit, AORN Antonio Cardarelli, Naples, Italy

Roberto Tarletti, SCDU Neurologia - Stroke Unit, Azienda Ospedaliero-Universitaria "Maggiore, Novara, Italy

Rossana Tassi, Urgency and Emergency Department -Azienda Ospedaliera Universitaria Senese, Siena, Italy

Tiziana Tassinari, Department of Neurology-Santa Corona Hospital, Pietra Ligure, Italy

Valeria Terruso, Department of Neurology, AOOR Villa Sofia-Cervello, Palermo, Italy

Pierluigi Tocco, Stroke Unit -"S.Spirito" Hospital, Pescara, Italy

Danilo Toni, Department of Human Neurosciences, University of Rome La Sapienza, Rome, Italy

Agnese Tonon, Department of Neurology, Ospedale Civile SS. Giovanni e Paolo, Venice, Italy

Laura Tudisco, Stroke Unit-Careggi University Hospital, Florence, Italy

Martina Valente, Stroke Unit-Department of Neurology, SS. Biagio e Arrigo, Alessandria. Italy

Maria Grazia Vittorini, Department of Biotechnological and Applied Clinical Sciences, University of L'Aquila, L'Aquila, Italy.

Gloria Valcamonica, Department of Neurology - ASST-Ovest Milanese, Legnano, Italy

Luisa Vinciguerra, Department of Neurology, ASST Crema Hospital, Cremona, Italy

Marco Vista, Unit of Neurology-San Luca Hospital, Lucca, Italy

Giovanna Viticchi, Experimental Medicine and clinical Department-Marche Polytechnic University, Ancona, Italy

Gino Volpi, Department of Neurology-San Jacopo Hospital, Pistoia, Italy

Marialuisa Zedde, Neurology Unit, Stroke Unit, Azienda Unità Sanitaria Locale-IRCCS di Reggio Emilia, Reggio Emilia

Mara Zenorini, Department of Neuroscience-Azienda Ospedaliera Universitaria Intergrata Verona, Verona, Italy

Andrea Zini, IRCCS Istituto delle Scienze Neurologiche di Bologna, Department of Neurology and Stroke Center-Maggiore Hospital, Bologna, Italy

Cecilia Zivelonghi, Department of Neuroscience-Azienda Ospedaliera Universitaria Intergrata Verona, Verona, Italy

Antonio Zito, Department of Cardiocerebrovascular diseases; Neurology-Stroke Unit-ASST Ospedale Maggiore di Crema, Crema, Italy
